# Ultrasonic-Controlled Drug Release Prevents Protumorigenic Transition and Improves Sequential Targeting Effect to Enhance Treatment of Residual Hepatocellular Carcinoma

**DOI:** 10.34133/bmr.0114

**Published:** 2025-01-29

**Authors:** Yongquan Huang, Songying Pi, Hui Chen, Shushan Zhang, Jianzhong Xian, Yuhong Lin, Jiaxing Chen, Qing Ye, Feile Ye, Yin Huang, Hailing Yu, Zhongzhen Su

**Affiliations:** ^1^ Department of Ultrasound, Fifth Affiliated Hospital of Sun Yat-sen University, Zhuhai, Guangdong Province 519000, China.; ^2^ Guangdong Provincial Key Laboratory of Biomedical Imaging and Guangdong Provincial Engineering Research Center of Molecular Imaging, Fifth Affiliated Hospital of Sun Yat-sen University, Zhuhai, Guangdong Province 519000, China.; ^3^ Center of Cardiovascular Disease, Fifth Affiliated Hospital of Sun Yat-sen University, Zhuhai, Guangdong Province 519000, China.

## Abstract

Insufficient radio-frequency ablation (IRFA) of hepatocellular carcinoma accelerates the recurrence of residual tumor, leading to a poor prognosis. Neutrophils (NEs), as the initial leukocytes to infiltrate the IRFA-associated inflammatory area, were utilized as drug carriers due to their inherent chemotactic properties for targeted delivery of chemotherapy drugs to the inflammatory site where residual tumor persists post-IRFA. Previous research has highlighted that the immunosuppressive cytokines in the tumor microenvironment could promote the transition of NEs into a protumorigenic phenotype. However, it is unclear whether NEs used as drug delivery carriers undergo similar changes and how this transition affects treatment effectiveness. Here, we present novel findings demonstrating the phenotypic transition of NEs in the residual tumor microenvironment from an antitumorigenic to a protumorigenic state following IRFA treatment. More critically, we found for the first time that NE carriers undergo a comparable phenotypic transition in the residual tumor, thereby attenuating the therapeutic outcome. Ingeniously, coloading NE carriers with perfluorohexane not only enabled ultrasound imaging but also facilitated spatiotemporally controllable drug release through ultrasound irradiation, thus preventing the protumorigenic transition of NE carriers and maintaining an inflammatory microenvironment at the residual tumor zone. This significantly improved the sequential targeting effect of NE carriers and ultimately enhanced the treatment of residual tumor post-IRFA. Our study provided novel insights into the modulatory role of the immune microenvironment on the phenotypic transition of live NE carriers in the drug delivery system and presented a strategy to prevent adverse effects and enhance residual tumor treatment.

## Introduction

The global incidence of primary liver cancer ranks sixth, while it stands as the third leading cause of cancer-related mortality worldwide. Hepatocellular carcinoma (HCC) represents the predominant type of liver cancer, accounting for approximately 75% to 85% of all cases [1]. Radio-frequency ablation (RFA) is a principal radical therapeutic approach for HCC, offering notable advantages in terms of safety, simplicity, rare adverse event rate, and reduced hospitalization duration [2]. In the process of RFA, elevated temperatures of up to 60 °C can induce coagulation necrosis of the ablated tissue. Regrettably, intricate spatial structures, indistinct tumor borders, and challenges in accessing the ablative margin give rise to insufficient radio-frequency ablation (IRFA) and residual tumor formation [3]. The local recurrence rate of HCC after RFA ranges from 2% to 36% [4]. Moreover, recurrent tumors often exhibit a higher malignant grade and lower sensitivity to conventional chemotherapy, ultimately leading to a dire prognosis [5–7]. Therefore, the eradication of residual tumor cells for suppressing tumor recurrence holds paramount importance in enhancing outcomes.

Therapeutic strategies utilizing endogenous leukocytes have been proposed as a strong potential strategy for targeted drug delivery [8–10]. Following RFA, an ablation-associated inflammatory response is elicited, characterized by the infiltration of leukocytes. Neutrophils (NEs), constituting 50% to 70% of circulating leukocytes in humans, are the initial immune cell population that promptly responds to inflammation and naturally navigates and penetrates the ablated lesion [11–13]. Based on this intrinsic characteristic, NEs have been proposed as cellular drug carriers for delivering agents to a range of inflammation-related disorders, such as residual tumor at the postoperative inflammatory site. This approach involves hitchhiking nanoparticles (NPs) with NEs or loading them into the cytoplasm to increase drug accumulation in the target lesions. Experimental confirmation has shown that NEs can respond to local signals at target sites and release intracellular drugs, thereby improving therapeutic efficacy [14]. The use of an exogenous stimulus–response mechanism represents a more efficient approach to drug release. However, it has not yet been established whether there is a difference in the efficiency of drug release and therapeutic effect between spontaneous and exogenous stimulus-induced releases in NE drug carriers.

In our previous research, we demonstrated that inflammatory cell infiltration into residual tumor leads to an activated immune response. Combining RFA with NE drug delivery may show a promising therapeutic strategy to benefit HCC patients. However, we further discovered such immune activation was transient and followed by a prominently suppressive immune microenvironment several days after IRFA [7]. It remains uncertain whether such rapid alternations in the immune microenvironment affect the sequential targeting of the live-cell carriers of NEs. Tumor cells and the immune microenvironment engage in intricate interactions, wherein tumor cell-released products interact to support NEs that play pivotal roles in the development and progression of HCC [15–17]. Previous studies have mainly focused on the inflammatory chemotaxis of NEs for drug delivery while neglecting the impact of the microenvironment on the cell carriers upon arrival at the tumor site. Additionally, it is widely acknowledged that infiltration of tumor-associated neutrophils (TANs) into the tumor environment can result in their transition into a protumorigenic phenotype [18–20], characterized by a spindle-shaped morphology, a prolonged lifespan, and delayed apoptosis, and ultimately contribute to an immunosuppressive environment and tumor progression [21,22]. A recent study postulated that the application of NEs as nanocarriers could potentially reprogram them to a protumorigenic state upon homing to tumor sites. To circumvent this potential impact, human pluripotent stem cells were genetically engineered into chimeric antigen receptor NEs, which exhibit a sustained antitumor phenotype even in immunosuppressive tumor microenvironments (TMEs), for glioblastoma chemoimmunotherapy [23]. However, it remains unconfirmed whether the immunosuppressive TME can induce such a phenotypic transition of NE carriers and consequently affect therapeutic efficiency.

**Fig. 1.**
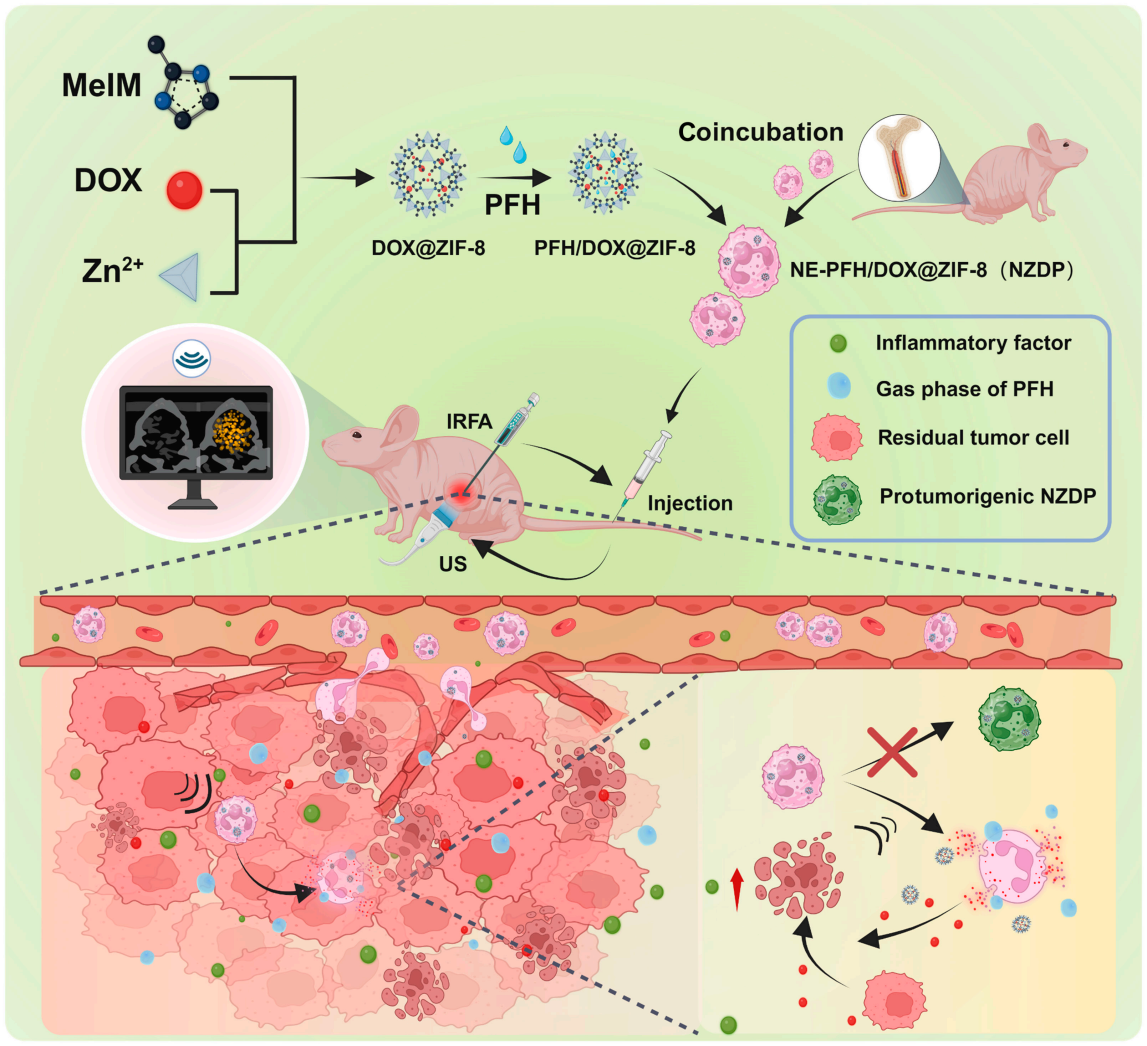
Schematic design of the synthesis of an NZDP drug delivery system in vitro and its application for residual tumor therapy post-insufficient radio-frequency ablation (post-IRFA) in vivo.

In this study, NEs were employed as drug carriers for targeted delivery of chemotherapy agents to eradicate residual tumor following IRFA while perfluorohexane (PFH) was concurrently incorporated into the NE carriers for ultrasound (US) imaging and triggered drug release. The impact of the immunosuppressive TME post-IRFA on the fate of the NE carriers and potential reduction of the therapeutic effects was investigated. Consequently, it was discovered for the first time that the immune microenvironment of residual tumor following IRFA delayed NE apoptosis and promoted the transition of TANs into a protumorigenic phenotype, thereby significantly impacting therapeutic efficacy. Furthermore, ultrasonic-controlled release of NE carriers could improve drug release efficiency to avoid these adverse effects and maintain a proinflammatory microenvironment at the residual tumor site. This promoted the sequential targeted recruitment of an NE drug delivery system, which in turn enhanced its antitumor effect post-IRFA (Fig. 1).

## Materials and Methods

### Cell lines and cell culture

Hepatoma cell lines (HepG2, HCC-LM3, and H22) had been previously acquired from the Cell Bank of the Chinese Academy of Sciences (Shanghai, China). The HepG2 and HCC-LM3 cells were grown in DMEM containing 10% fetal bovine serum and 1% penicillin/streptomycin double antibody. The H22 cells were grown in RPMI 1640 containing 10% fetal bovine serum and 1% penicillin/streptomycin double antibody. All cell lines were incubated in a humidified atmosphere of 5% CO_2_ at 37 °C.

### Isolation of NEs

The Percoll gradient method was used for the isolation of NEs from the bone marrow of mice. Briefly, mice were euthanized to extract the tibia and femur, followed by centrifugation and resuspension to obtain bone marrow cells. Sequential addition of 2 ml each of 78%, 65%, and 55% Percoll solution was performed, with a final addition of 2 ml of bone marrow cell solution. NEs were harvested from the 78% and 65% interfaces. Flow antibodies (PE-conjugated CD11b and FITC-conjugated Ly-6G) were used to detect NE purity. Flow cytometry was employed to evaluate the drug-loading capacity of NEs. Y585-PE-A was selected as the flow channel.

### Preparation of ZDP (ZIP) and NZDP (NZIP)

First of all, 200 mg of zinc nitrate hexahydrate and 2 g of dimethylimidazole were dissolved in 1 and 10 ml of double-distilled water, respectively. Subsequently, 4 ml of DOX or ICG aqueous solution with a concentration of 10 mg/ml was added dropwise to the zinc nitrate hexahydrate solution, followed by the addition of the dimethylimidazole solution to the mixture of zinc nitrate hexahydrate and DOX or ICG under ice bath conditions. After centrifugation and vacuum freeze-drying, DOX@ZIF-8 (ZD) or ICG@ZIF-8 (ZI) NPs were harvested. PFH/DOX@ZIF-8 (ZDP) or PFH/ICG@ZIF-8 (ZIP) NPs were obtained by injecting PFH into ZD or ZI powder under vacuum conditions. NZDP or NZIP was obtained by coincubating the isolated NEs and prepared ZDP for 1 h.

### Characterization

Transmission electron microscopy (TEM) was employed to verify the morphology and elemental components of ZIF-8, ZD, and ZDP NPs. X-ray diffraction (XRD) was performed to determine their crystalline structures. Ultraviolet–visible (UV–vis) spectroscopy was utilized to obtain UV–vis absorption spectra. Measurement of zeta potential was conducted using a laser nanometer instrument (Zetasizer Nano ZSE, Malvern, UK). Confocal laser scanning microscopy (Zeiss 880, Zeiss, Germany) was employed for recording fluorescence images of cells.

### US-controlled DOX release from NZDP

The NZDP solutions were agitated on a horizontal shaker at 100 r/min. At predetermined intervals, 1 ml of solution was sampled and replaced with phosphate-buffered saline (PBS) while maintaining the original volume during shaking. The DOX content in the supernatant was quantified using a UV–vis spectrophotometer.

### US-enhanced imaging of NZDP in vivo

Tumor-bearing mice were intravenously injected with NZDP (5 × 10^5^ cells) or PBS post-IRFA. Subsequently, US was used to irradiate the tumor site (1 W/cm^2^, 5 min) after injection, and both B-mode and contrast-enhanced US images of the tumor site were recorded before and after irradiation.

### Cell viability assay

Cell Counting Kit-8 (CCK-8; DOJINDO, Kumamoto, Japan) was adopted to detect NE or tumor cell viability. Tumor cells or NEs were seeded into 96-well plates at a density of 5 × 10^3^ cells per well. Subsequently, tumor cells were treated with NEs, NEs + US, ZIF-8, US, NZDP, or NZDP + US, respectively. In addition, NEs were exposed to varying DOX doses of ZDP or DOX for different durations. Then, the cells were cultured with the medium containing 10% Cell Counting Kit-8 solution. A multimode reader (Synergy HTX, Bio-Rad, USA) was used to record the absorbance value at 450 nm, and cell viability was calculated.

### Inherent chemotaxis of NZDP in vitro

The inflammation-targeting ability of NZDP was investigated by Transwell migration assay. Briefly, NEs or NZDP was seeded into the upper chambers of Transwell inserts (Transwell polycarbonate membrane: 3-μm pore size, 6.5-mm diameter, and 0.33-cm^2^ membrane surface area, Corning) at a density of 1 × 10^2^ cells per well in serum-free RPMI 1640 medium. The lower chambers were added with different concentrations of tumor necrosis factor-alpha (TNF-α) for varying durations. Cells that migrated to the lower side of the Transwell membranes were stained with crystal violet and photographed under a light microscope, and the number of migrated cells and the chemotactic index were quantified.

### Inherent chemotaxis of NZDP in vivo

The IRFA subcutaneous tumor model was established as previously described [4]. The inflammation-targeting ability of NZDP was recorded with an in vivo imaging system (IVIS Lumina, PerkinElmer, USA). Tumor-bearing mice were injected with NZIP, ZIP, or ICG via the tail vein post-IRFA. Subsequently, the mice were anesthetized with isoflurane and imaged at various time points. Experiments were performed to verify the sequential inflammation-targeting ability of NZDP combined with US administration after different treatments (IRFA, US post-IRFA, NZDP post-IRFA, or NZDP combined with US irradiation post-IRFA). Then, the mice received a tail vein injection of NZIP and underwent imaging at different time points using an in vivo imaging system.

### Drug permeability in 3-dimensional tumor spheroids

The H22 cells were seeded into low-adhesion 6-well plates at a density of 5 × 10^2^ per well and cultured with DMEM/F12 medium supplemented with EGF and β-FGF. After culturing for 14 d, the tumor spheroids were harvested and incubated with DOX, ZDP, or NZDP for 8 h. Images were obtained using Z-stack tomoscanning (confocal laser scanning microscopy; Zeiss 880, Zeiss, Germany).

### Establishment of tumor-bearing mice

Healthy BALB/c nude mice aged 4 to 5 weeks were adaptively raised for 1 week and then injected subcutaneously to form tumors. Before injection, the skin was disinfected with 75% alcohol. The amount of H22 cells injected into each mouse was 10 × 10^4^ (100 ml of PBS). After injection, the injection site was gently pressed for 15 s, and the local skin was disinfected with alcohol again. The basic condition of the mice was closely observed, and IRFA was performed when the tumor volume grew to 600 to 800 mm^3^.

### Establishment of IRFA mouse model

The mice were anesthetized and their skin was disinfected, and then the mice were placed prone on a VIVA grounding pad. A radio-frequency needle was inserted into about one-third of the long axis of the tumor under US guidance. In order to achieve the purpose of IRFA, the output power of the radio-frequency transmitter was 5 W and the time was 20 s. Finally, the mice were closely observed and moved back to the breeding room to wait for administration.

### In vivo therapeutic effect

Mice treated with IRFA were administered (PBS, DOX, NZDP: 5 × 10^5^ cells, equivalent to 25 μg of DOX, NZDP + US: 5 × 10^5^ cells, equivalent to 25 μg of DOX, 1 W/cm^2^, 5 min) on the first and third days post-IRFA, respectively. To evaluate the therapeutic effect, tumor volume was recorded using the following formula: volume = (width^2^ × length)/2. Blood samples were collected for biochemical tests, and vital organs were removed for staining on day 14.

### Apoptosis assay

NEs were incubated with the supernatant of unheated tumor cells and sublethal heated tumor cells for varying durations, followed by collection and staining with annexin V–FITC and PI solutions for the evaluation of apoptosis. The apoptosis rate was determined based on the flow cytometry results.

### Statistical analysis

Each experiment was performed 3 times independently, and quantitative data were expressed as mean ± standard deviation. Statistical analysis was performed using GraphPad Prism 7.0. For comparison between 2 groups, a *t* test or a nonparametric test was performed after assessing normality. For experiments with more than 3 groups, normality and variance were assessed followed by either analysis of variance or a rank-sum test. *P* value correction was subsequently applied, and a statistically significant difference between groups was defined as *P* < 0.05.

## Results

### IRFA promotes the transition of TANs into a protumorigenic phenotype and induces an immunosuppressive TME

**Fig. 2.**
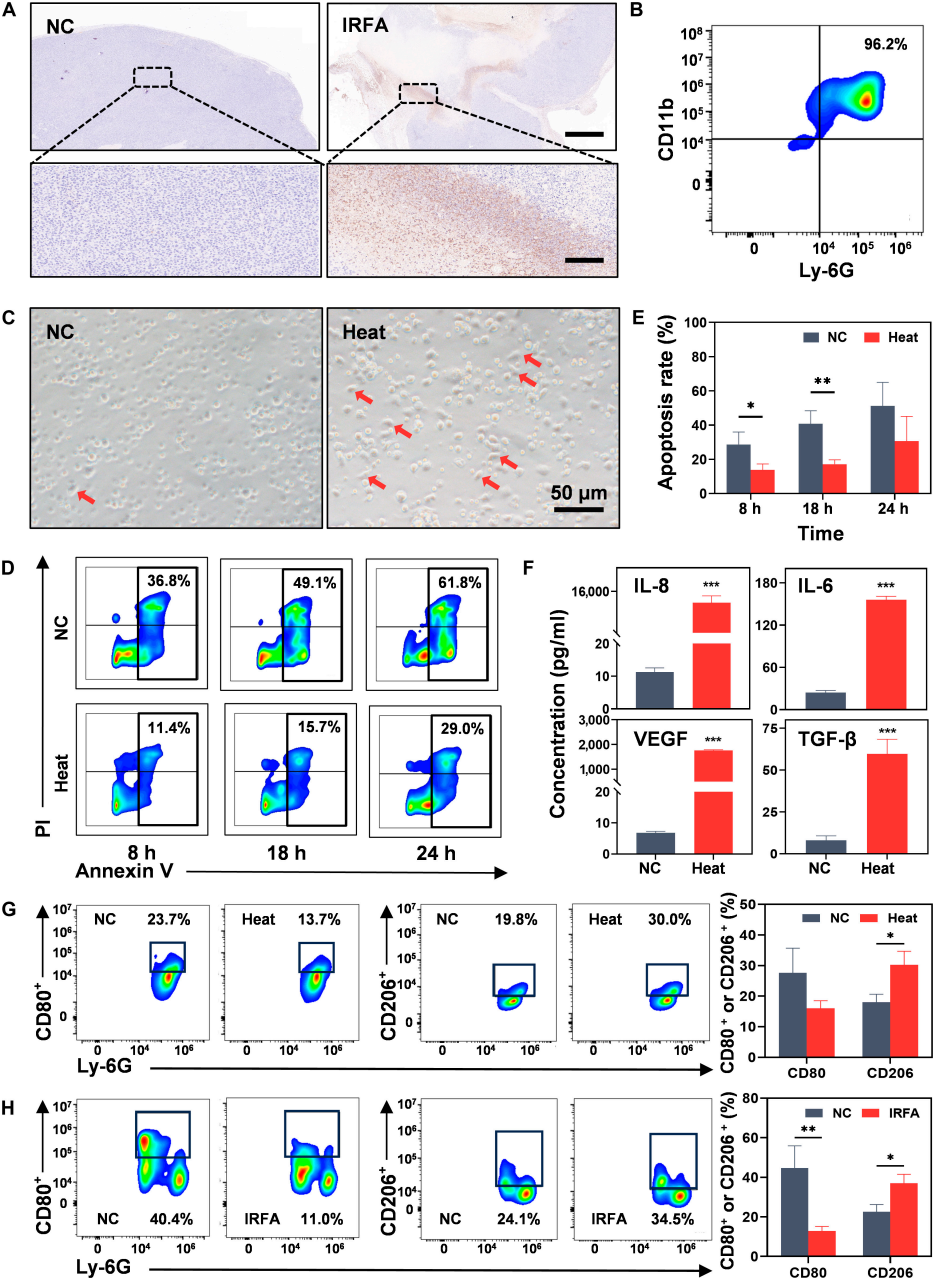
IRFA promotes tumor-associated neutrophils’ (TANs’) protumorigenic phenotype transition and immune suppression. (A) Ly-6G immunohistochemical staining of the tumor before and after IRFA. Scale bars: 1,000 μm (upper), 100 μm (lower). (B) Flow cytometric analysis of neutrophils (NEs) stained with fluorescein isothiocyanate (FITC)-conjugated Ly-6G and PE-conjugated CD11b antibodies. (C) Morphological changes of NEs after coincubation with the supernatant from a sublethal heating model. Arrow: spindle-shaped NEs; scale bar: 50 μm. (D and E) Flow cytometric analysis of the apoptosis rate of NEs after coincubation with the supernatant of a sublethal heating model (D) and quantification (E). *n* = 3. (F) Levels of interleukin-8 (IL-8), interleukin-6 (IL-6), vascular endothelial growth factor (VEGF), and transforming growth factor-beta (TGF-β) after coincubation of NEs and the supernatant from a sublethal heating model. *n* = 4. (G) The levels of CD80^+^ and CD206^+^ NEs after coincubation with the supernatant from the sublethal heated hepatocellular carcinoma (HCC) cells or unheated HCC cells in vitro. *n* = 3. (H) The levels of CD80^+^ and CD206^+^ NEs before or after IRFA in vivo. *n* = 3. *P < 0.05; **P < 0.01; ***P < 0.001.

To investigate whether IRFA promotes NE recruitment, an IRFA mouse model was established. The results demonstrated that IRFA significantly promoted infiltration of NEs into the residual tumor, as shown by both Ly-6G immunohistochemical staining (Fig. 2A) and hematoxylin and eosin staining (Fig. S1). It has been reported that infiltrating macrophages within residual tumor can transition into a protumorigenic phenotype, thereby promoting tumor progression and impeding immunotherapy [24]. However, it has not been studied whether the residual tumor environment after IRFA induces the phenotypic transition of the infiltrating NEs. To unravel this mystery, NEs were isolated from mouse bone marrow and purified using density-gradient centrifugation. The purity was quantified to be as high as 96.2% by flow cytometer analysis (Fig. 2B). Subsequently, isolated NEs were coincubated with the supernatant of sublethal heated HCC cells. Morphologically, more spindle-shaped NEs were observed after coincubation for 24 h compared to those in the control group, where most NEs maintained a circular shape (Fig. 2C). The morphological changes in NEs suggested an increased potential for protumorigenic phenotype following coincubation [22]. Apoptosis is considered the predominant cell death pathway in NEs [25]. Recent evidence has confirmed that the TME facilitates the recruitment and survival of TANs. The extended lifespan of TANs may be a critical factor in the contribution of NEs to tumor progression and metastasis [26]. Here, the apoptotic effect of the TME on the recruited NEs post-IRFA was explored. Flow cytometry analysis revealed that NEs incubated with the sublethal HCC cell supernatant exhibited apoptosis rates of 13.8% ± 3.6%, 17.2% ± 2.6%, and 30.7% ± 14.4% at 8, 18, and 24 h, respectively, while NEs without coincubation showed higher apoptosis rates of 28.7% ± 7.3%, 40.8% ± 7.6%, and 51.2% ± 13.8% at the corresponding time points (Fig. 2D and E). The results showed that NEs, when coincubated with the sublethal HCC cell supernatant, caused a delay in apoptosis, acting as a potent immunosuppressive signal within the TME [13]. Subsequent enzyme-linked immunosorbent assay (ELISA) detection also demonstrated a remarkable increase in secretion of cytokines such as interleukin-6 (IL-6), interleukin-8, vascular endothelial growth factor, and transforming growth factor-beta following incubation, all of which played crucial roles in immunosuppression (Fig. 2F). The expression of low CD80^**+**^ levels and high CD206^**+**^ levels of NEs is considered a characteristic feature of protumorigenic TANs [27]. Further phenotypic analysis consistently confirmed the results by showing that coincubation reduced the ratio of CD80^+^ NEs from 27.6% ± 8.1% to 16.0% ± 2.5% while increasing the ratio of CD206^+^ NEs from 18.1% ± 2.6% to 30.3% ± 4.4% in vitro (Fig. 2G). Similarly, IRFA decreased the CD80^+^ TAN ratio from 44.6% ± 11.3% to 12.9% ± 2.3% while raising the ratio of CD206^+^ TANs from 22.6% ± 3.7% to 37.0% ± 4.5% in vivo (Fig. 2H). Taken together, these results indicated that a large number of NEs can spontaneously chemotax to the residual tumor area where the inflammatory response was elicited following IRFA. Furthermore, residual tumor cells influenced NEs to undergo delayed apoptosis and a protumorigenic phenotypic conversion. These changes significantly increased the development of an immunosuppressive microenvironment, which could potentially reduce the acute-inflammatory chemotaxis of NEs.

### Preparation and characterization of NZDP

**Fig. 3.**
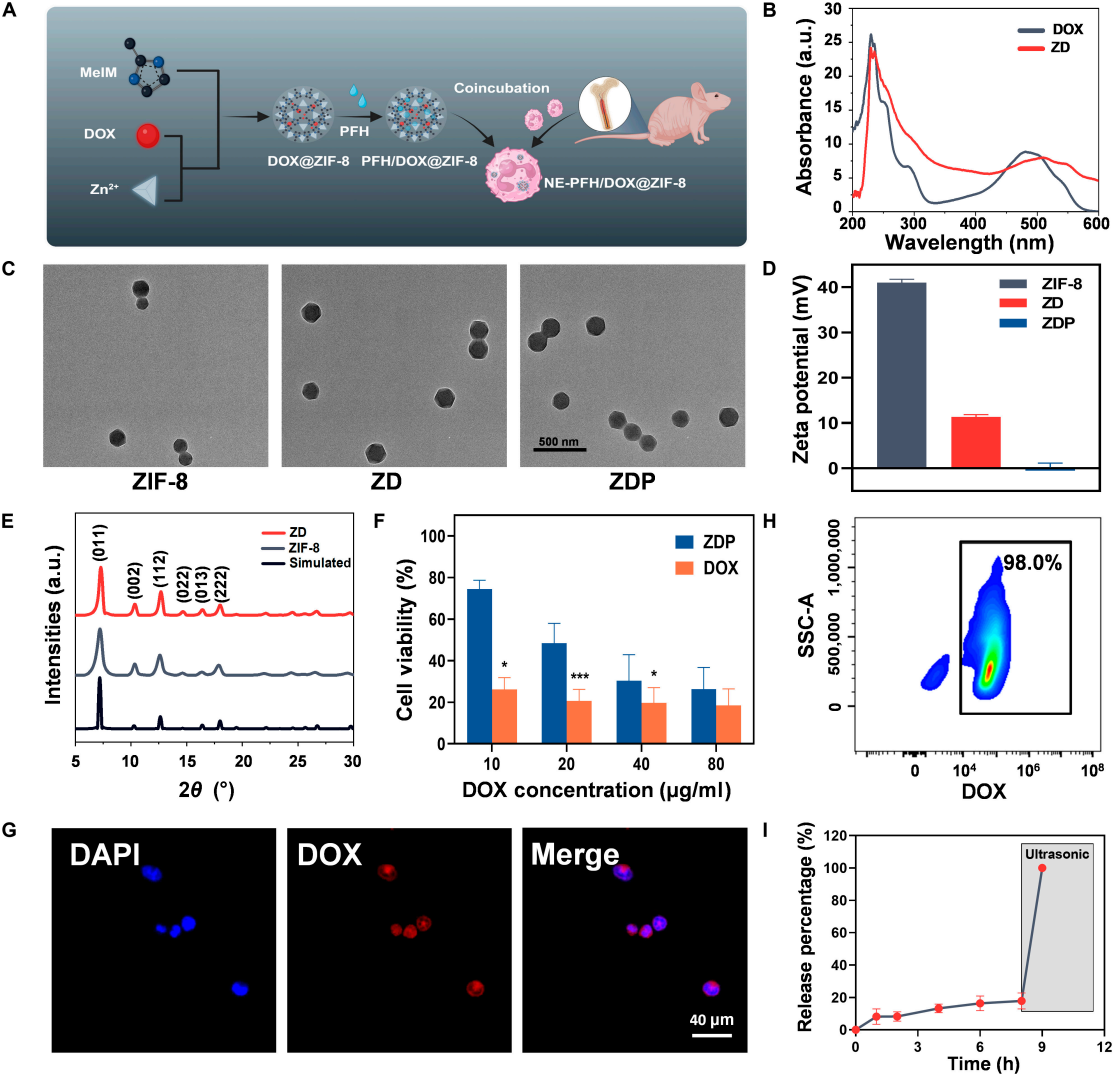
Preparation and characterization of NZDP. (A) The synthetic procedure of NZDP. (B) Ultraviolet–visible (UV–vis) spectroscopy of free DOX and DOX@ZIF-8 (ZD). (C) Transmission electron microscopy (TEM) images of ZIF-8, ZD, and PFH/DOX@ZIF-8 (ZDP). Scale bar: 500 nm. (D) Zeta potentials of ZIF-8, ZD, and ZDP. (E) X-ray diffraction (XRD) patterns of ZD, ZIF-8, and simulated ZIF-8. (F) Cytotoxicity of free DOX or ZDP with various concentrations against NEs. *n* = 3. (G) Fluorescence microscopy images of NZDP. Scale bar: 20 μm. (H) Flow cytometric analysis of the drug-loading capacity of NZDP. (I) Drug release of NZDP in vitro. *n* = 3. *P < 0.05; ***P < 0.001. PFH, perfluorohexane; DAPI, 4′,6-diamidino-2-phenylindole.

NEs have been utilized as drug delivery carriers due to their inherent inflammatory chemotaxis and have shown significant therapeutic effects in inhibiting postoperative residual tumor [8,9]. However, the potential impact of the residual TME on the protumorigenic transition of NE-based drug delivery systems to therapeutic efficacy remains unknown. To address this question, NEs were employed as drug carriers to deliver DOX and PFH was coloaded to induce rapid drug release by sonication, thereby avoiding NE retention in the TME of the residual tumor. The drug delivery system mediated by NEs was constructed as shown in Fig. 3A. DOX was encapsulated into the framework of ZIF-8 named DOX@ZIF-8 (ZD) using the one-pot method [28]. UV–vis spectroscopy analysis revealed an absorption peak at approximately 505 nm for ZD, which was a typical peak of DOX but with a 15-nm redshift, indicating successful loading of DOX (Fig. 3B). Subsequently, freeze-dried ZD was infused with PFH under vacuum conditions to prepare PFH/DOX@ZIF-8 (ZDP). TEM analysis confirmed the successful fabrication of polyhedral ZIF-8 NPs and presented the morphology details of ZD and ZDP NPs (Fig. 3C). The average hydrodynamic diameters of ZIF-8, ZD, and ZDP were 144.7, 317.7, and 321.2 nm, respectively (Fig. S2). The zeta potentials of ZIF-8, ZD, and ZDP were 41.0 ± 0.8, 11.3 ± 0.5, and 0.2 ± 1.0 mV, respectively (Fig. 3D). The positive charge on the surface of ZIF-8 NPs can be attributed to the presence of Zn^2+^ vacancies. After drug loading, the amino groups on the DOX molecules can establish coordination interactions with Zn^2+^, thereby effectively neutralizing the positive charge present on the surface of ZIF-8 and consequently reducing the electrostatic potential. This observation further corroborates the successful encapsulation of DOX. In addition, the negative charge carried by fluorine atoms due to their strong oxidizing property may explain the additional reduction in potential after loading PFH. The XRD pattern confirmed that the characteristic peaks of the prepared NP materials, ZIF-8 and ZD, closely matched those of the simulated ZIF-8. The result indicates that the crystal structure of ZIF-8 remained intact after modification (Fig. 3E). NZDP was obtained by incubating NEs with ZDP, which encapsulated various concentrations of DOX. It was observed that NZDP maintained good cell viability of 74.5% ± 4.2% at a DOX concentration of 10 μg ml^−1^ for 1 h and retained 71.0% ± 10.5% viability even after 6 h of coincubation. In contrast, free DOX exhibited extreme cytotoxicity against NEs (Fig. 3F and Fig. S3), indicating that loading of DOX on ZIF-8 significantly reduced its toxicity to NE carriers. The successful preparation of NZDP was visually confirmed through fluorescence microscopy images showing cytoplasmic loading of DOX within NEs (Fig. 3G), which achieved a high drug-loading rate of up to 98.0% (Fig. 3H and Fig. S4). Then, drug release behavior analysis of NZDP was conducted at different time points. The results revealed that more than 80% of the DOX remained in NZDP for up to 8 h in the absence of sonication, while complete release occurred immediately upon sonication (Fig. 3I). In conclusion, a smart NE-mediated drug delivery system capable of ultrasonic control was synthesized. By loading PFH, this system may exhibit the ability to avoid retention in residual tumor and enable US imaging.

### US imaging of NZDP

**Fig. 4.**
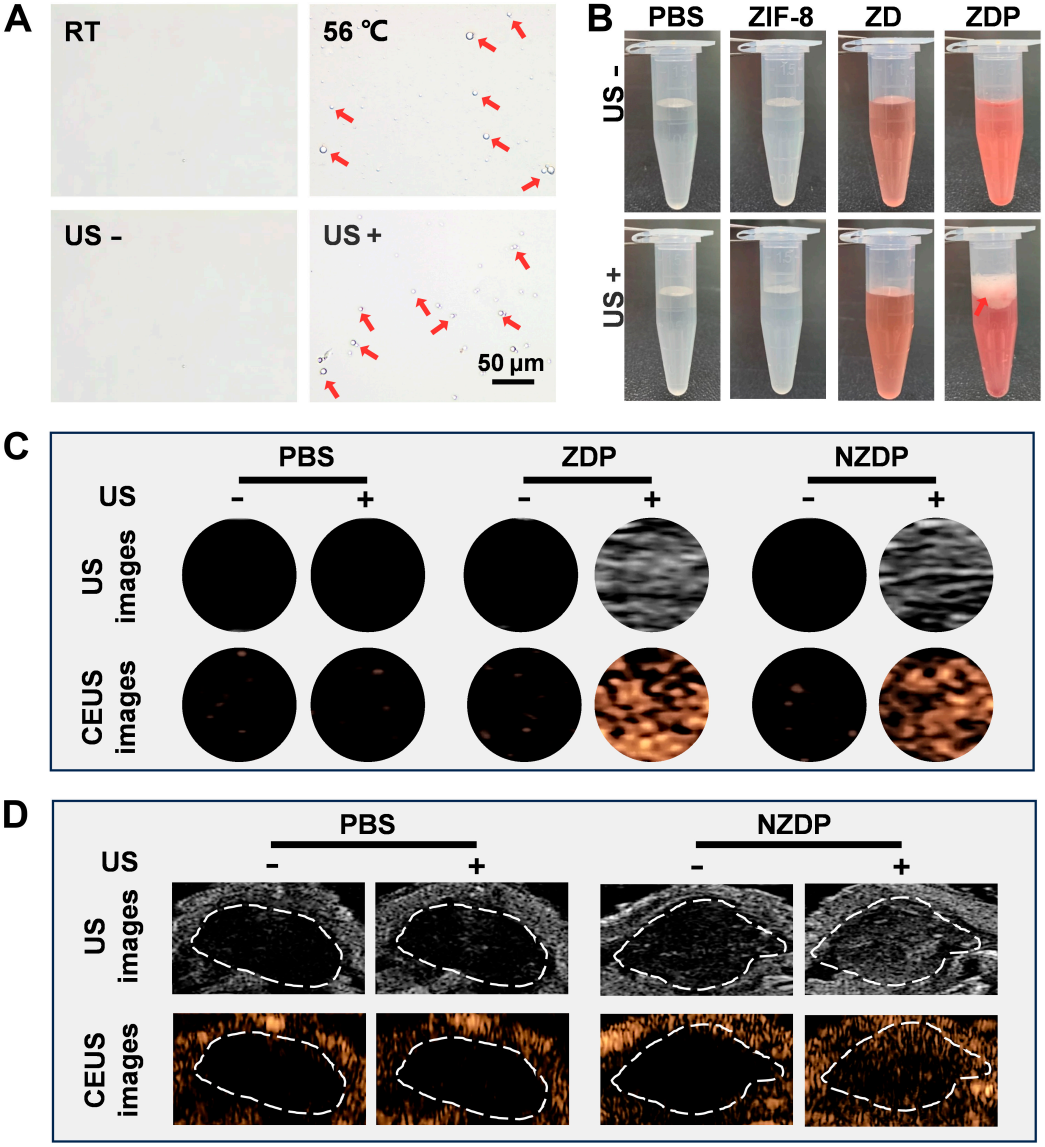
Ultrasound (US) imaging of NZDP. (A) Microscopic pictures of ZDP before and after heating (56 °C) and sonication (3 W/cm^2^, 2 min). Arrow: bubbles. Scale bar: 50 μm. (B) Details of bubble formation in phosphate-buffered saline (PBS), ZIF-8, ZD, and ZDP with or without sonication (3 W/cm^2^, 2 min). (C) B-mode and contrast-enhanced ultrasound (CEUS) images of PBS, ZDP, and NZDP with or without sonication (3 W/cm^2^, 2 min) in vitro. (D) B-mode and CEUS images of PBS and NZDP with or without sonication (1 W/cm^2^, 5 min) in a subcutaneous tumor model. RT, room temperature.

The ability of the constructed NZDP to undergo a phase transition from liquid to gas state and facilitate visually controlled drug release was further confirmed. ZDP was activated using a low-frequency US instrument and subjected to heat treatment under an optical microscope. Upon exposure to heat treatment at 56 °C or sonication at 3 W/cm^2^ for 2 min, numerous bubbles were observed as depicted in Fig. 4A. Notably, compared with the PBS, ZIF-8, and ZD groups, the ZDP group exhibited a large production and accumulation of bubbles above the liquid in the tube after exposure to sonication at 3 W/cm^2^ for 2 min (Fig. 4B). The US imaging properties of ZDP and NZDP were evaluated using B-mode and contrast-enhanced ultrasound (CEUS) techniques in vitro with or without sonication exposure. As shown in Fig. 4C, no significant changes were observed in either the B-mode or CEUS images in the PBS group with or without sonication irradiation. By contrast, both ZDP and NZDP showed significantly improved signal intensities after sonication exposure in both B-mode and CEUS mode due to the sonication-mediated liquid–gas phase transition of PFH induced by US. These results revealed that both ZDP and NZDP could facilitate the visualization of bubbles following sonication. Interestingly, before sonication, incubation of NZDP at 37 °C did not exhibit an obvious bubble echo, suggesting the inherent stability of NZDP in vitro. This observation can be attributed to the excellent stability of PFH at 37 °C, which is lower than the phase transition temperature of 56 °C. Encouraged by the promising US imaging performance of NZDP in vitro, we proceeded to assess its capability for ultrasonography in vivo. To select the appropriate conditions of liquid–gas phase transition in vivo, we referred to research and selected similar experimental conditions to conduct experiments [29,30]. Consistent with the findings in vitro, no visible bubble formation was observed in the tumor region prior to sonication. However, following sonication, NZDP demonstrated an enhanced imaging effect within the tumor area (Fig. 4D). This observation indicates that NZDP maintained stability upon reaching the tumor site until it received ultrasonic stimulation, which resulted in the generation of a significant number of bubbles, thereby enhancing US imaging. These results suggest that NZDP is a suitable US imaging agent for effectively guiding the complete ultrasonic-controlled release of drugs in the tumor area.

### Inherent chemotaxis and optimal permeability of NZDP increase its drug accumulation at the residual tumor post-IRFA

**Fig. 5.**
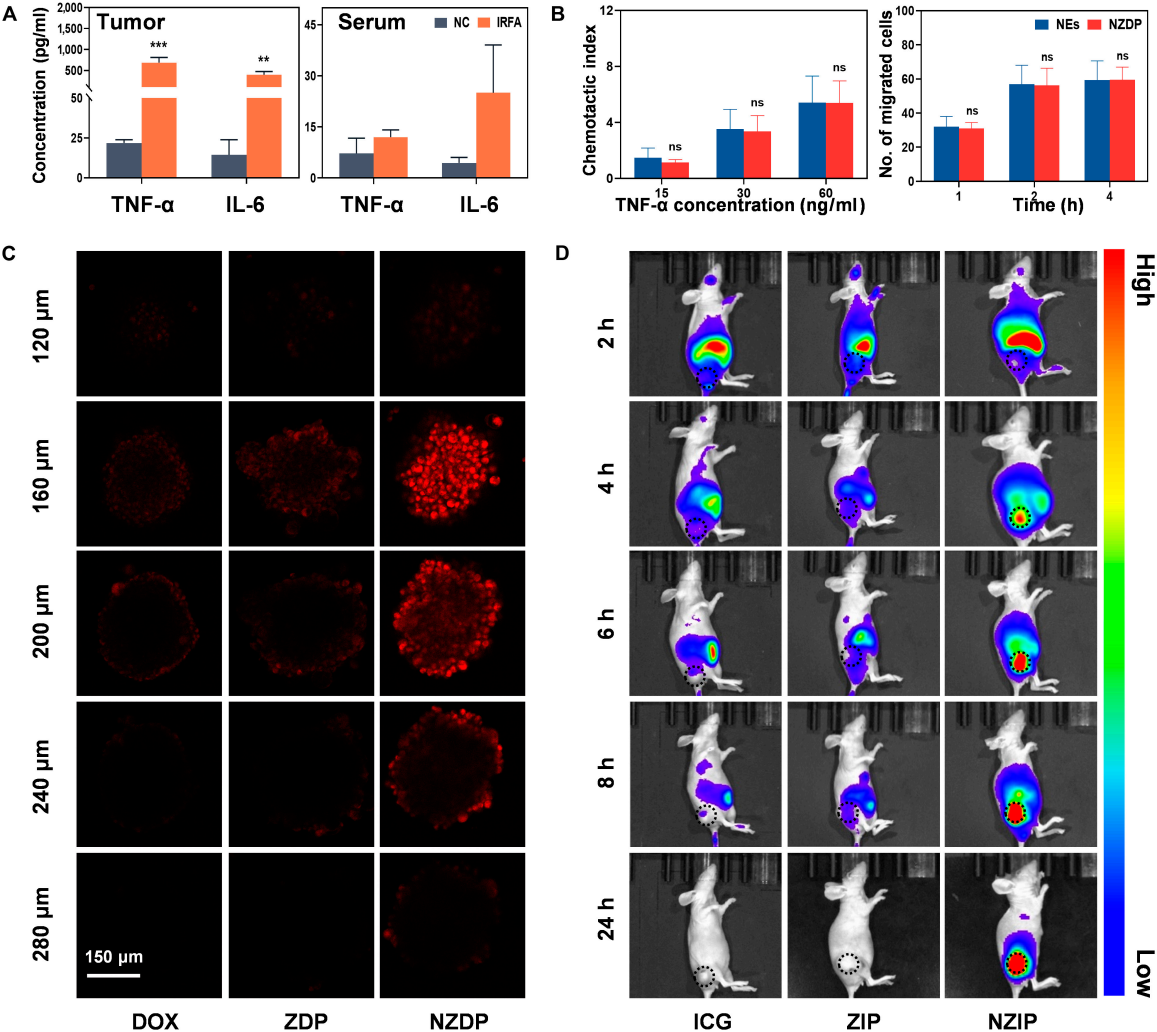
Properties of chemotaxis and permeability make NZDP suitable for the treatment of residual tumor post-IRFA. (A) Levels of tumor necrosis factor-alpha (TNF-α) and IL-6 in residual tumor and serum before or 12 h after IRFA. *n* = 3. (B) Impact of TNF-α concentration or duration on the chemotactic ability of NEs or NZDP. *n* = 3. (C) Tumor permeability of free DOX, ZDP, or NZDP with 3-dimensional tumor spheroids. Confocal laser scanning microscope images were obtained with a Z-stack thickness of 40 μm. Scale bar: 150 μm. (D) Fluorescence images in vivo of tumor-bearing mice after intravenous injection of ICG, ZIP, or NZIP at 2, 4, 6, 8, and 24 h. **P < 0.01; ***P < 0.001. ns, no statistically significant difference.

NEs were verified as efficient carriers for targeting the inflammatory site based on their inherent chemotaxis properties [13]. To assess the impact of IRFA on inflammatory signals and the subsequent tumor-targeting ability of NEs, ELISAs were employed to measure cytokine concentrations 12 h post-IRFA. The results showed that IRFA significantly up-regulated TNF-α and IL-6 at the residual tumor site by 31-fold and 28-fold compared with NC, respectively. However, there was no obvious increase observed in the serum concentrations (Fig. 5A and Fig. S5). This created an intense cytokine concentration gradient to form a favorable microenvironment for NZDP chemotaxis. Furthermore, the Transwell migration assay confirmed that NZDP presented increasing chemotaxis to TNF-α in a concentration-dependent and time-dependent manner, comparable to that of NEs (Fig. 5B and Fig. S6). These findings suggested that NZDP retained the physiological activities of NEs, enabling them to actively respond to inflammatory stimuli and migrate toward the inflamed sites following IRFA in vitro. Considering the limited deep penetration of general chemotherapy agents into residual tumor, a 3-dimensional tumor spheroid model was employed to investigate the tumor permeability of NZDP. Following an 8-h incubation period, a strong red fluorescence of DOX was distributed throughout most areas of the spheres in the NZDP group, whereas only a weak signal could be detected at the periphery of the spheroids in both the DOX and ZDP groups (Fig. 5C). Semiquantitative analysis showed significant differences in fluorescence intensity between NZDP and ZDP, as well as between NZDP and free DOX (Fig. S7). These findings demonstrated that encapsulating ZDP into the NE carriers retained the excellent tumor permeability of NEs in vitro. Next, an ICG-labeled NE delivery system was synthesized to explore the distribution in vivo based on its active chemotaxis and excellent permeability. The fluorescence signal at the residual tumor site was observed at the specified time point after injection via the tail vein. As shown in Fig. 5D and Fig. S8, a stronger fluorescence signal appeared in the residual tumor area of the NE-PFH/ICG@ZIF-8 (NZIP) group 4 h postinjection, which significantly increased throughout 24 h. However, no accumulation of fluorescent signals in the tumor area was seen in the other groups during the detection period. The present finding vividly demonstrated that residual tumor post-IRFA could elicit an inflammatory response to facilitate the chemotactic migration of NZDP. The properties of inherent chemotaxis and optimal permeability of NE carriers may increase drug accumulation at the residual tumor site, potentially leading to improved therapeutic outcomes.

### US-controlled release of NZDP enhances antitumor cell efficacy in vitro

**Fig. 6.**
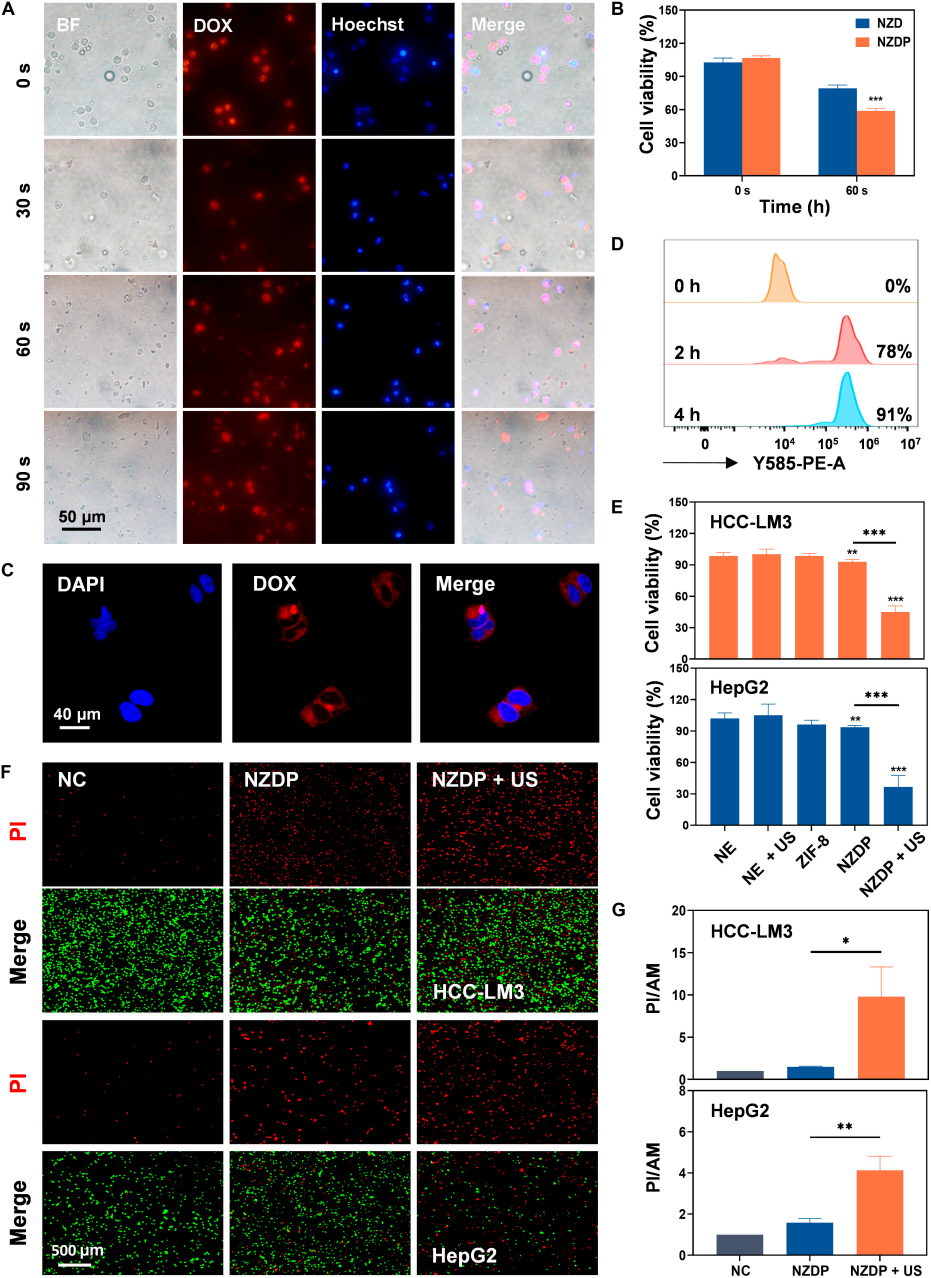
US-controlled release of NE-carrier-mediated drug enhances antitumor cell efficacy in vitro. (A) Morphological changes in NE carriers and DOX release after sonication for 0, 30, 60, and 90 s. Scale bar: 50 μm. (B) Impact of sonication for different durations on the viability of HCC cells treated with NZD or NZDP. *n* = 3. (C) Fluorescence images of DOX within tumor cells. Scale bar: 40 μm. (D) Drug uptake efficiency by tumor cells. (E) Effects of NEs, NEs + US, ZIF-8, NZDP, or NZDP + US on the activity of HCC-LM3 cells (upper) and HepG2 cells (lower). *n* = 3. (F and G) Living/dead cell double staining of HCC-LM3 cells (upper) and HepG2 cells (lower) (F) treated with PBS, NZDP, or NZDP + US and quantification (G); scale bar: 500 μm. *n* = 3. *P < 0.05; **P < 0.01; ***P < 0.001.

The application of sonication was employed to modulate drug release by inducing targeted carrier disruption, capitalizing on its deep tissue penetration and minimal impact on normal tissues. PFH loaded with NE carriers undergoes a liquid–gas phase transition following ultrasonic irradiation. The generation of microbubbles and rapid changes in volume contribute to the disruption of the NE carriers, facilitating the effective release of the drug. This approach aimed to further investigate the potential influence of the TME following IRFA of the NE carriers. As shown in Fig. 6A, the NE carriers exhibited a pronounced drug distribution throughout the cytoplasm before US treatment. The phase transition of PFH was triggered by applying sonication of varying durations, resulting in the release of the payload encapsulated within the NEs. With prolonged ultrasonic irradiation, the number of microbubbles generated by the phase change of PFH gradually diminished. Concurrently, the NE carriers underwent progressive shape deformation and lysis, leading to extracellular drug release. In comparison to the PFH-unloaded group (79.2% ± 3.0%), only 59.0% ± 1.8% of NEs loaded with PFH survived after sonication (Fig. 6B). Furthermore, NEs loaded with PFH resulted in a significantly enhanced drug release rate of approximately 4.1 ± 1.7 times after sonication for 60 s (Fig. S9). These results presented that ultrasonic treatment effectively disrupts NEs by promoting the phase transition of PFH, thereby enabling spatiotemporally controllable drug release from the NZDP drug delivery system. Subsequently, the culture supernatant from the control group or the irradiated NZDP group was coincubated with HCC cells for 24 h to validate the inhibitory effect of DOX released from sonicated NZDP on tumor cell proliferation. Strong fluorescence of DOX was observed within the tumor cells after coincubation (Fig. 6C), indicating successful drug delivery into the tumor cells. Drug absorbing rates at 2 and 4 h were 78% and 91%, respectively (Fig. 6D). Furthermore, US-induced release of DOX from NZDP resulted in a significant inhibition of cell viability against HCC-LM3 and HepG2 tumor cells, with 55% and 63% inhibition, respectively. In contrast, NZDP without sonication exhibited only minimal cell viability inhibition (6% and 7%) (Fig. 6E). Similarly, double staining of living/dead cells revealed that sonicated NZDP demonstrated superior efficacy in eradicating HCC cells compared to the other groups (Fig. 6F and G). These findings strongly indicated that ultrasonic stimulation of NE carriers could enable controlled drug release and facilitate efficient therapeutic outcomes.

### US-controlled release of NZDP enhances the antiresidual tumor efficacy post-IRFA

**Fig. 7.**
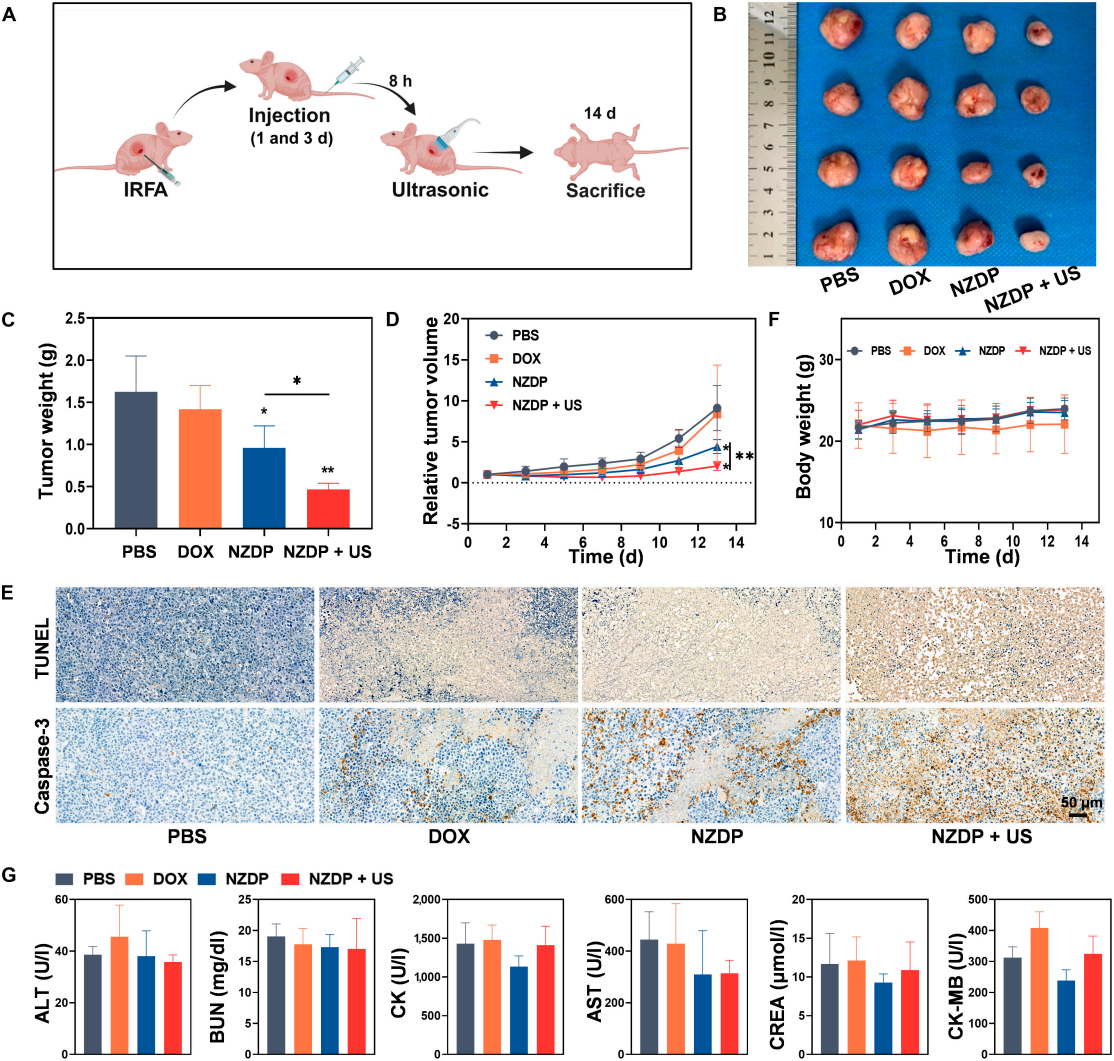
US-controlled release of NZDP enhances the antiresidual tumor effect post-IRFA. (A) Treatment procedure post-IRFA. (B) Ex vitro images of residual tumor after PBS, DOX, NZDP, or NZDP + US treatment. (C) Residual tumor weight of each group. (D) Changes in relative tumor volume in the residual tumor during treatments. (E) TUNEL (upper) and caspase-3 (lower) staining of residual tumor after different treatments. Scale bar: 50 μm. (F) Weight changes of each group during treatments. (G) Levels of ALT, BUN, CK, AST, CREA, and CK-MB in mouse blood after different treatments. *n* = 4. *P < 0.05; **P < 0.01.

Encouraged by the promising results in vitro, we further evaluated the antitumor efficacy of NZDP in vivo and assessed whether the TME induced a potential influence on the NE carriers. To ensure efficient drug release without retention of NZDP in the TME, a mild low-frequency US irradiation at 1 W/cm^2^ for 5 min was employed to induce PFH vaporization. The therapeutic schedule is outlined in Fig. 7A. Tumor volumes and weights were recorded at the designated time point. The results showed that the therapeutic efficacy of both the NZDP and NZDP + US groups significantly suppressed the growth of tumor. Interestingly, the combination of NZDP and sonication demonstrated a more efficacious inhibition of tumor growth, achieving a 70.98% reduction in tumor volume, which was 1.74 times greater than that of the NZDP groups alone (Fig. 7B to D). In addition, the free DOX group exhibited limited efficacy against tumors at equivalent therapeutic concentrations, potentially due to inadequate tumor tissue penetration and subsequent insufficient drug concentration [31]. Tunnel analysis and caspase-3 staining also consistently demonstrated that the NZDP combined with the sonication group exhibited enhanced caspase-3 activation and increased tumor cell apoptosis compared to the other groups (Fig. 7E). Moreover, there were no significant differences observed in the body weights among all treatment groups (Fig. 7F). Subsequently, to evaluate the biocompatibility of each treatment, the main organs from the mice in each group were subjected to hematoxylin and eosin staining, and histological changes were evaluated at the end of the experiment. Simultaneously, blood samples were collected for biochemical analysis. Compared to the PBS group, no notable alterations in the major organs and no evident abnormalities in blood biochemical indicators were found in DOX, NZDP, or NZDP + US groups (Fig. S10 and Fig. 7G). These results demonstrated that US combined with the NZDP drug delivery system exhibited excellent biocompatibility. To summarize, our findings validated that sonication enhanced the efficacy of NZDP in inhibiting residual tumor growth in vivo. This can be attributed to the rapid release of DOX and prevention of NZDP retention at the residual tumor site through ultrasonic stimulation. However, further elucidation is warranted to understand the underlying reasons for the differential therapeutic outcomes observed with or without sonication.

### US-controlled release of NZDP prevents the protumorigenic transition of NE carriers and improves the sequential targeting effect

**Fig. 8.**
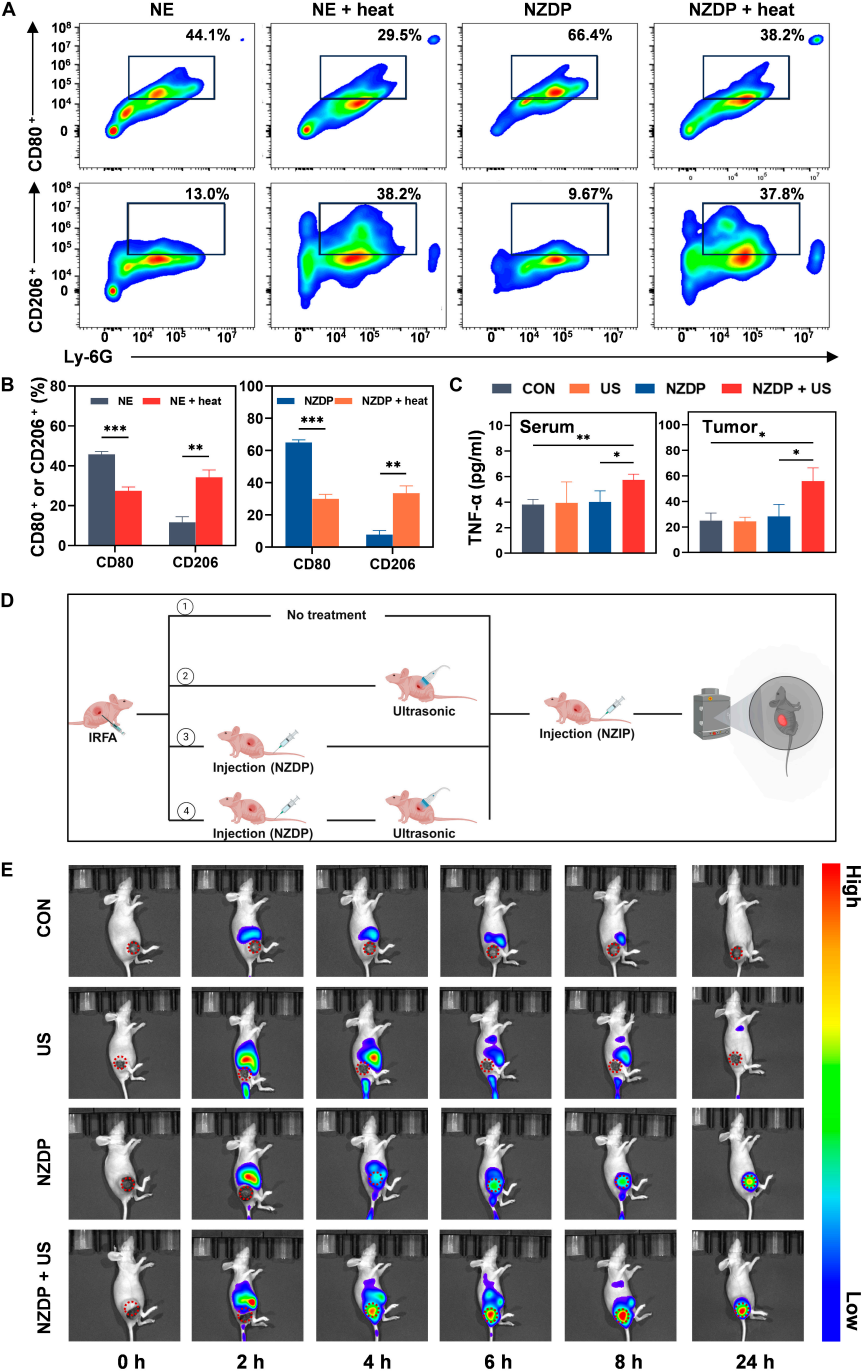
US-controlled release of NZDP prevents the protumorigenic phenotype transition of NE carriers and improves the sequential targeting effect. (A and B) Flow cytometric analysis of the levels of CD80^+^ and CD206^+^ NEs and NZDP coincubation with or without the supernatant of the sublethal heating model (A) and quantification (B). *n* = 3. (C) Levels of TNF-α in residual tumor and serum in each group. *n* = 3. (D and E) Schematic diagram of different treatments after IRFA and fluorescence images in vivo of tumor-bearing mice with different treatments after intravenous injection of NZIP at 0, 2, 4, 6, 8, and 24 h (E). *P < 0.05; **P < 0.01; ***P < 0.001.

The immune microenvironment plays a critical in determining the therapeutic outcomes of tumors [32,33]. Our previous findings have suggested that the TME post-IRFA could significantly induce a protumorigenic transition of TANs. Further examinations were conducted to investigate whether the immune-suppressive TME leads to a similar phenotypic transition of NZDP and contributes to the limited treatment efficacy. The in vitro microscopy results, similar to those of NEs, demonstrated that the drug-loaded NE carriers also transitioned into a spindle shape after coincubation with the supernatant from the sublethal heated HCC cells (Fig. S11). Next, it was further analyzed whether drug-loaded NZDP would undergo phenotypic transition similar to that of NEs after coincubation with the supernatant from the sublethal heated tumor cells. Flow cytometry analysis showed a decrease in CD80^**+**^ expression from 64.9% ± 1.7% to 30.0% ± 2.8% and an increase in CD206^**+**^ expression from 7.8% ± 2.6% to 33.6% ± 4.5% of drug-loaded NZDP after coincubation, which was similar to the trends of NEs (Fig. 8A and B). These findings suggested a protumorigenic transition within the NE carriers of the drug delivery system. The phenotypic transition may ultimately lead to delayed apoptosis of NE carriers, thereby serving as a potent immunosuppressive signal. Further ELISA test results confirmed that the combination of NZDP and sonication post-IRFA remained an augmented inflammatory response at residual tumor areas in vivo compared to the NZDP treatment alone. Additionally, in comparison with the NZDP group, the TME exhibited more pronounced inflammatory characteristics following the combined treatment of NZDP combined with sonication, as evidenced by elevated levels of TNF-α cytokines at the tumor site (Fig. 8C). This phenomenon can be attributed to the controlled release of NE carriers through ultrasonic irradiation, which effectively prevents delayed apoptosis and protumorigenic transition of NE carriers and results in a more proinflammatory response within the residual tumor. Furthermore, this desired inflammatory microenvironment may ultimately facilitate the continuous homing of NZDP and ultimately enhance tumor cell eradication. This hypothesis was validated through the designed experimental scheme (Fig. 8D). Concretely, the ICG-labeled NE delivery system was employed to investigate the drug distribution in vivo, aiming to evaluate whether the enhanced inflammatory response resulting from the initial treatment of NZDP combined with sonication could enhance the targeting efficiency of subsequent injection. The results showed that the fluorescence intensity was detectable at the residual tumor site post-IRFA in both the NZDP and NZDP + US groups. However, the NZDP + US group exhibited a significantly higher fluorescence intensity as early as 2 h, which continued to increase over time. In contrast, notable fluorescence intensity was detected only at 4 h in the NZDP group. Ultimately, it was found that the combined US group showed more effective fluorescence enrichment in the residual tumor area compared to other groups (Fig. 8E and Fig. S12). In addition, the CON group was imaged at the same time point after other groups were processed, instead of the NZIP group in Fig. 5D that was imaged after IRFA. At this time, the inflammation level in the residual tumor has been shown to have decreased in previous results, which may be the reason why NE carriers cannot be enriched in the CON group. This is why we need to combine US and NE carriers to increase the level of inflammation in the residual tumor for treatment. Based on the aforementioned results, the application of US-controlled release of NZDP effectively circumvented the protumorigenic transition of NE carriers and improved the sequential targeting effect, which eventually induced an additional therapeutic outcome surpassing that achieved by NZDP alone.

## Conclusion

In summary, we constructed an NE-mediated drug delivery system, which was capable of US imaging and spatiotemporally controllable drug release through sonication for the treatment of residual tumor post-IRFA. For the first time, we detected that TANs underwent a transition from an antitumorigenic to a protumorigenic phenotype in the immunosuppressive microenvironment elicited by IRFA. Importantly, our study revealed that the NE carriers of the live-cell drug delivery system also experienced a similar fate of transitioning into a protumorigenic phenotype, which attenuated their subsequent therapeutic efficacy. By utilizing sonication to trigger drug release and avoid retention of the NE carriers, we prevented such a transition and maintained an inflammatory microenvironment at the residual tumor site, thereby improving their sequential targeting effect. This approach efficiently enhanced the treatment of residual tumor post-IRFA. Our study provided a novel insight into how the microenvironment influenced the fate of NE-mediated drug delivery systems and their therapeutic efficacy. This observation may have implications for other immune cellular drug delivery systems as well, where rapid drug release in response to external stimulation can be adopted as a strategy to prevent potential adverse effects.

## Data Availability

Additional data related to this article may be available from the corresponding author on reasonable request.
