# Supplementary material for: Ultrasonic-Controlled Drug Release Prevents Protumorigenic Transition and Improves Sequential Targeting Effect to Enhance Treatment of Residual Hepatocellular Carcinoma
Source: Biomater Res. 2025 Jan 29;29:0114. doi: 10.34133/bmr.0114 (PMC11775379; doi:10.34133/bmr.0114)
Supplement: Supplementary 1 — Figs. S1 to S12 [file bmr.0114.f1.docx]

*Supplementary Material*

Ultrasonic-controlled drug release prevents pro-tumorigenic transition and improves sequential targeting effect to enhance treatment of residual HCC

*Yongquan Huang ^ab, 1^, Songying Pi ^ab, 1^, Hui Chen ^ab, 1^, Shushan Zhang ^a^, Jianzhong Xian ^a^, Yuhong Lin ^a^, Jiaxing Chen ^a^, Qing Ye ^a^, Feile Ye ^a^, Yin Huang ^c, *^, Hailing Yu ^b, *^, Zhongzhen Su ^a, *^*

^a^ Department of Ultrasound, Fifth Affiliated Hospital of Sun Yat-sen University, Zhuhai, Guangdong Province, 519000, China.

^b^ Guangdong Provincial Key Laboratory of Biomedical Imaging and Guangdong Provincial Engineering Research Center of Molecular Imaging, Fifth Affiliated Hospital of Sun Yat-sen University, Zhuhai, Guangdong Province, 519000, China.

^c^ Center of Cardiovascular Disease, Fifth Affiliated Hospital of Sun Yat-sen University

^1^ These authors contributed equally

^*^ Corresponding authors:

huangyin3@sysu.edu.cn （Yin Huang）

yuhling3@mail.sysu.edu.cn （Hailing Yu）

suzhzh3@mail.sysu.edu.cn （Zhongzhen Su）


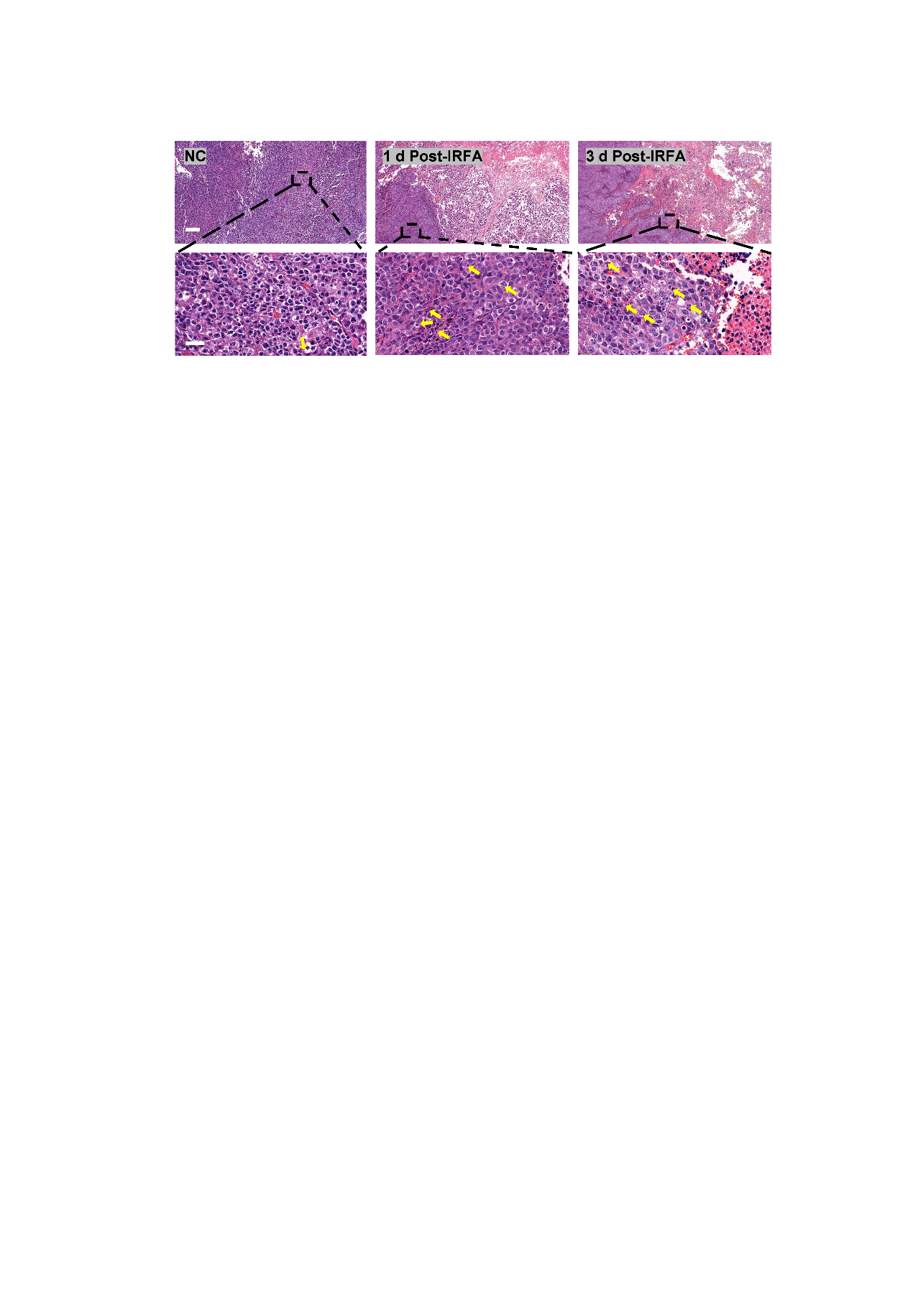


**Figure S1**. H&E staining before and 1 d, 3 d post-IRFA. Arrow: NEs. Scale bar: 200 μm (upper), 40 μm (lower).


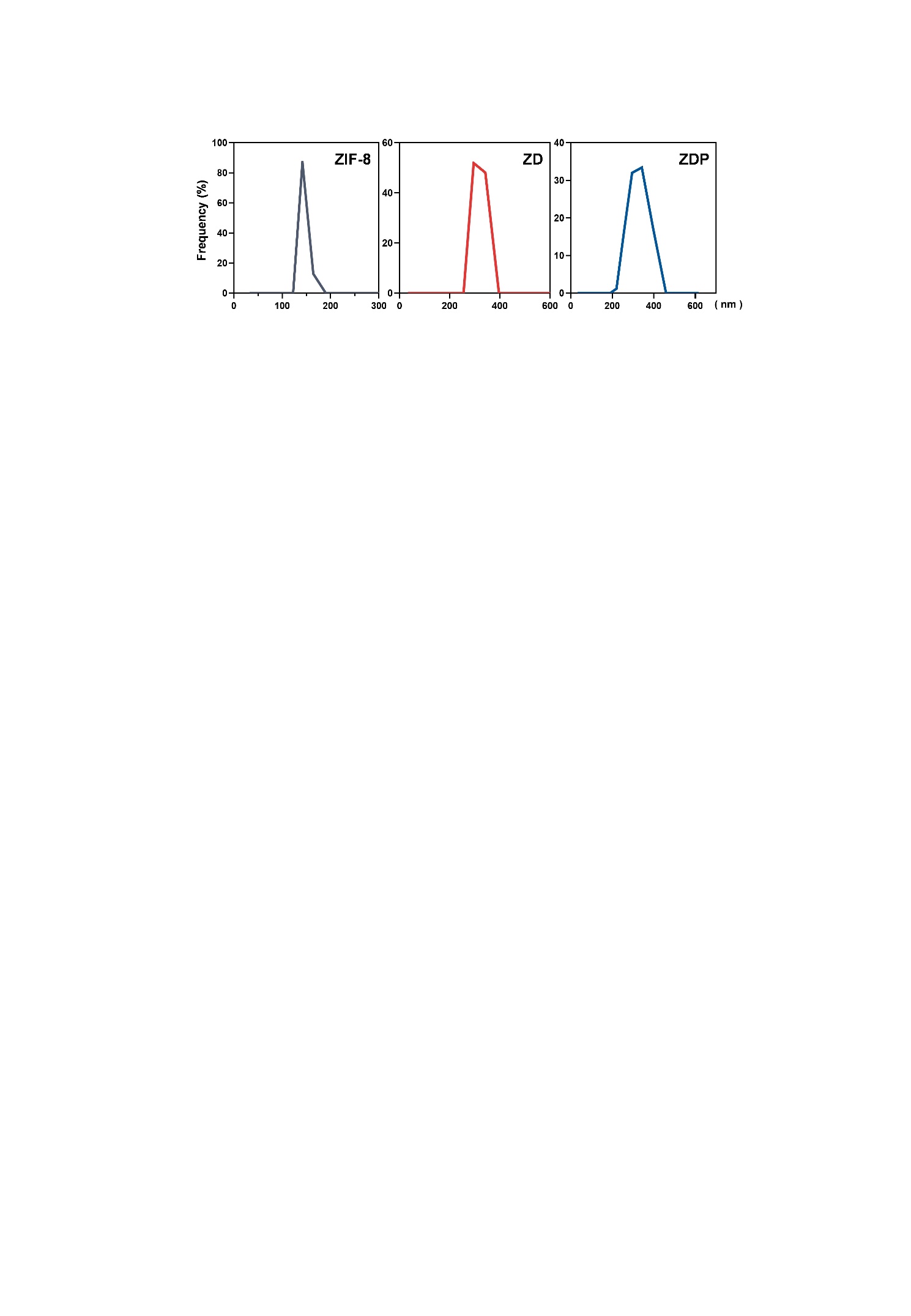


**Figure S2**. The hydrodynamic diameters of ZIF-8, ZD, and ZDP.


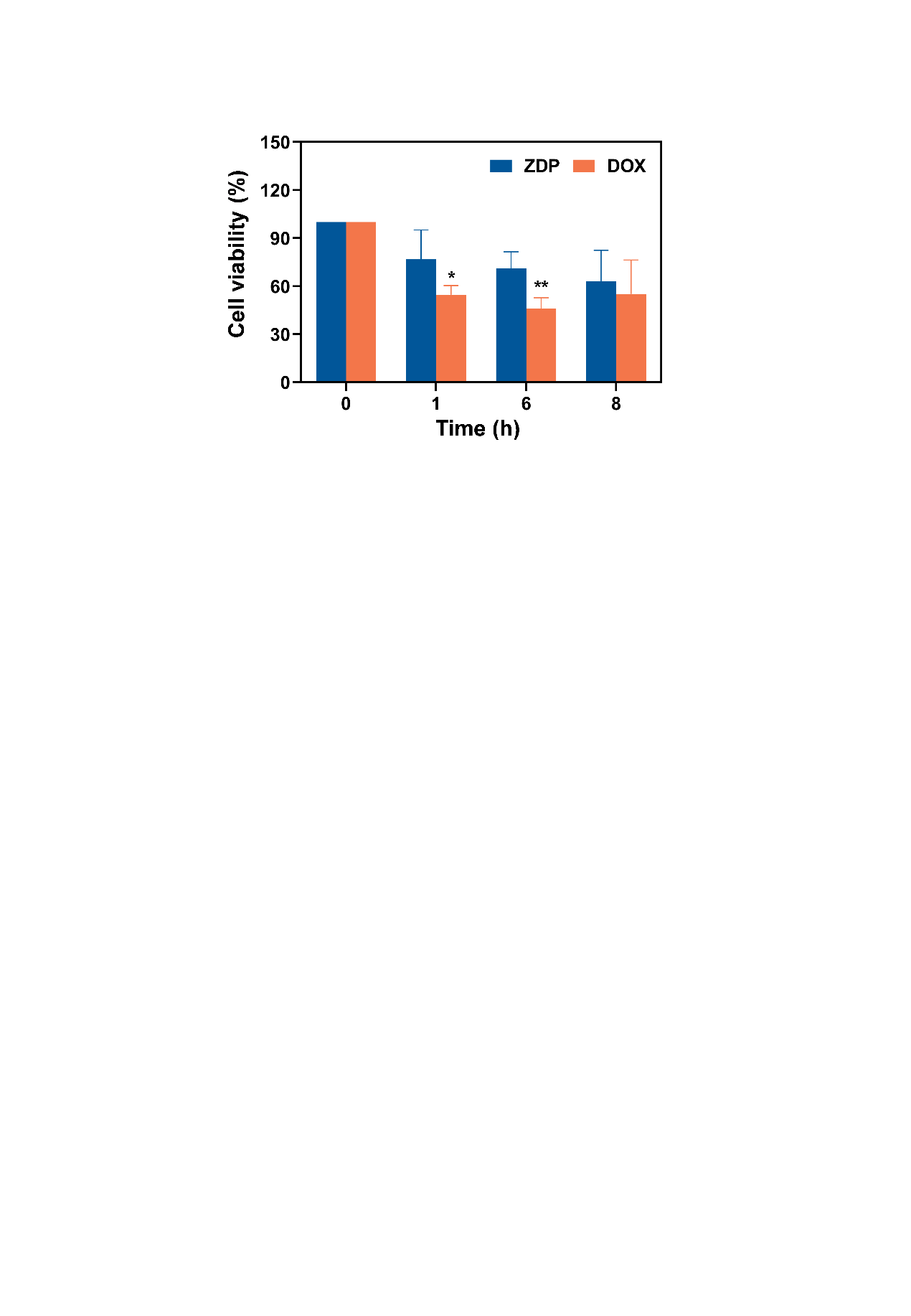


**Figure S3**. Cytotoxicity of free DOX and ZDP against NEs after different times. n = 3; **P*＜0.05; ***P*＜0.01.


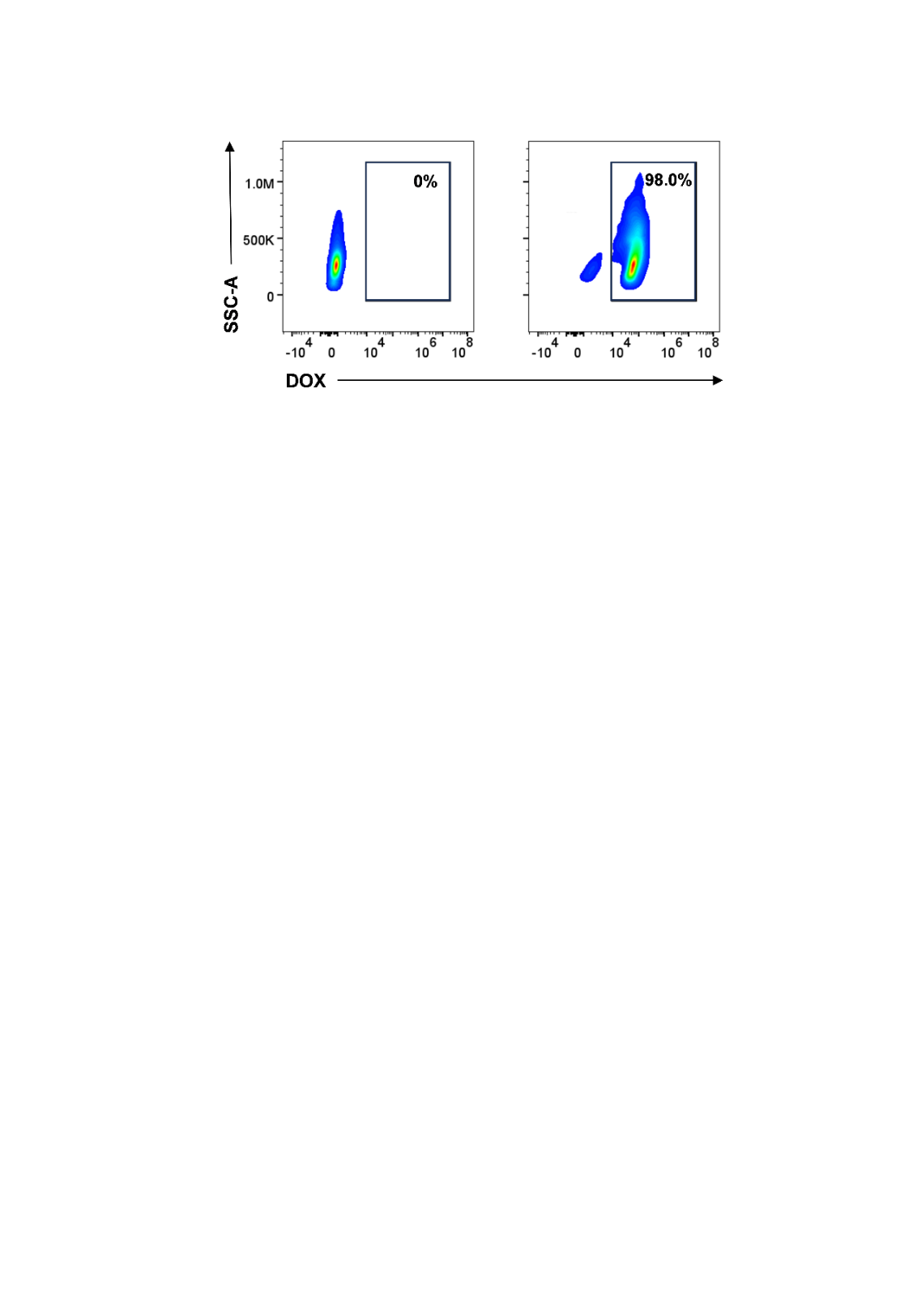


**Figure S4**. Flow cytometry analysis of NE before and after co-incubation with ZDP respectively. DOX fluorescence signal was monitored via the PE-A channel.


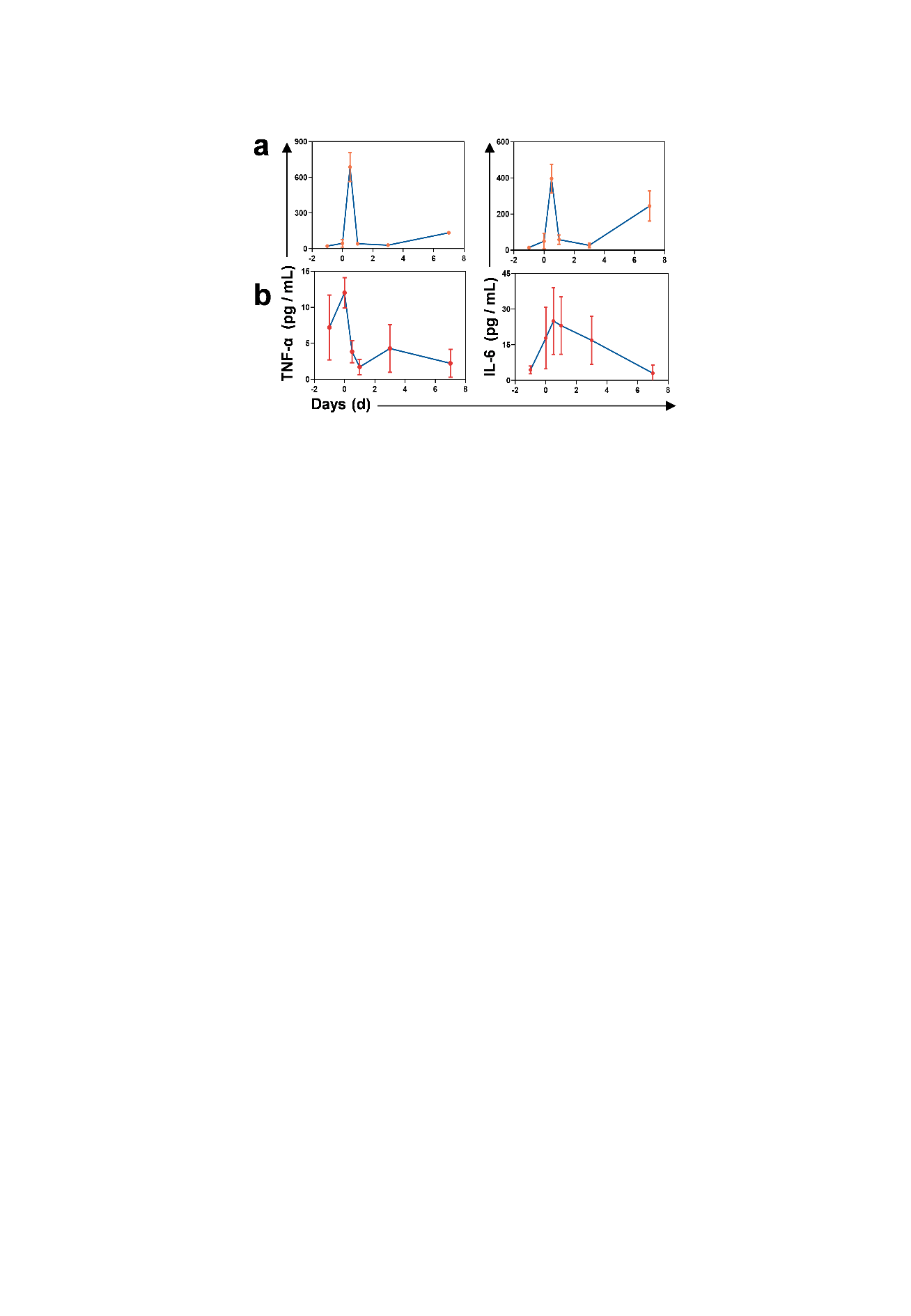


**Figure S5**. The inflammation factor levels of tumor and serum after IRFA. (a) Changes in TNF-α and IL-6 levels in tumor before and after IRFA. (b) Changes in TNF-α and IL-6 levels in serum before and after IRFA.


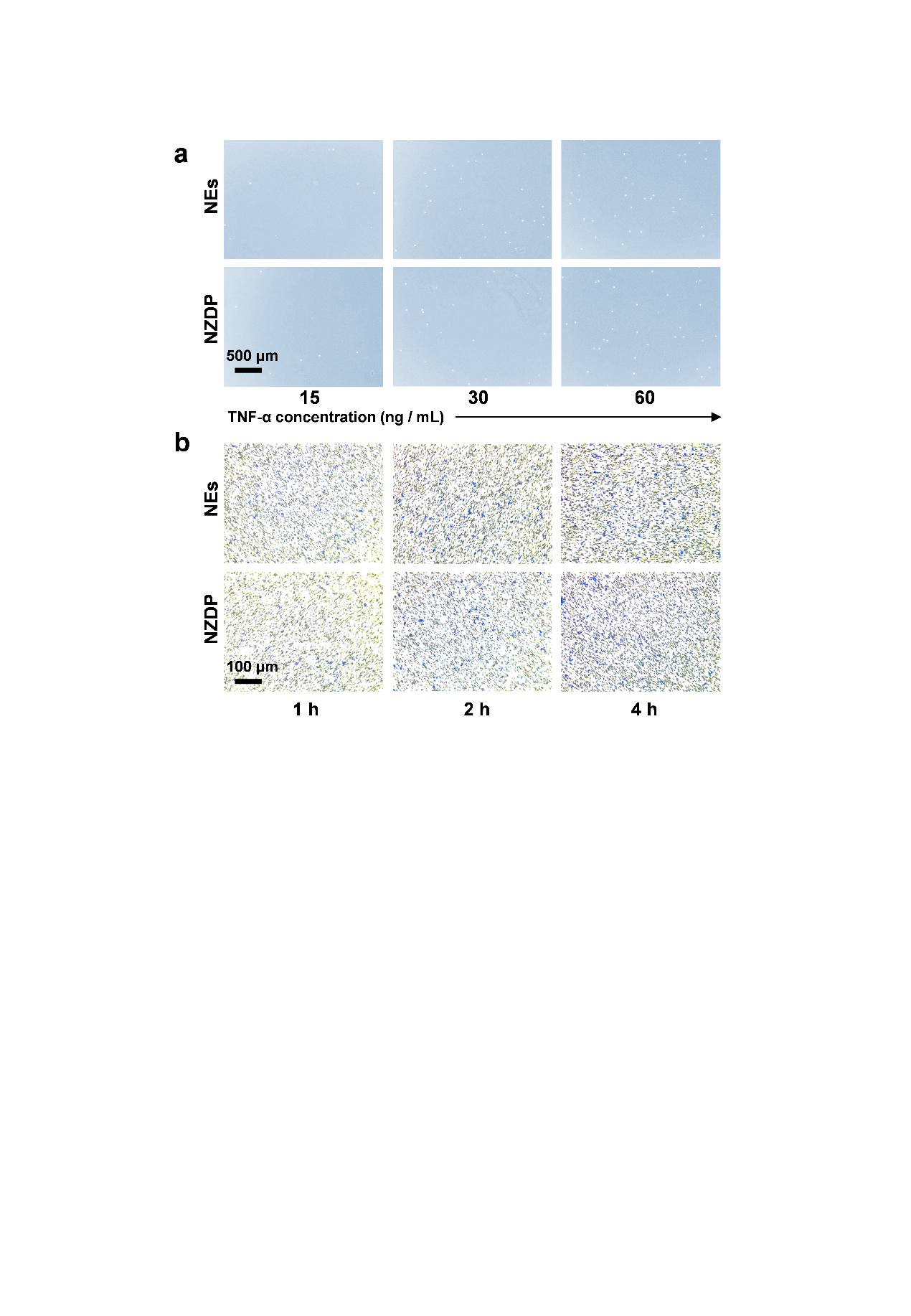


**Figure S6**. Effects of TNF-α concentration and time on the chemotactic ability of NEs and NZDP. (a) Pictures of Transwell lower chamber from each group under the microscope. (b) Pictures of Transwell NC membrane from each group under the microscope. Scale bar: 500 μm (upper), 100 μm (lower).


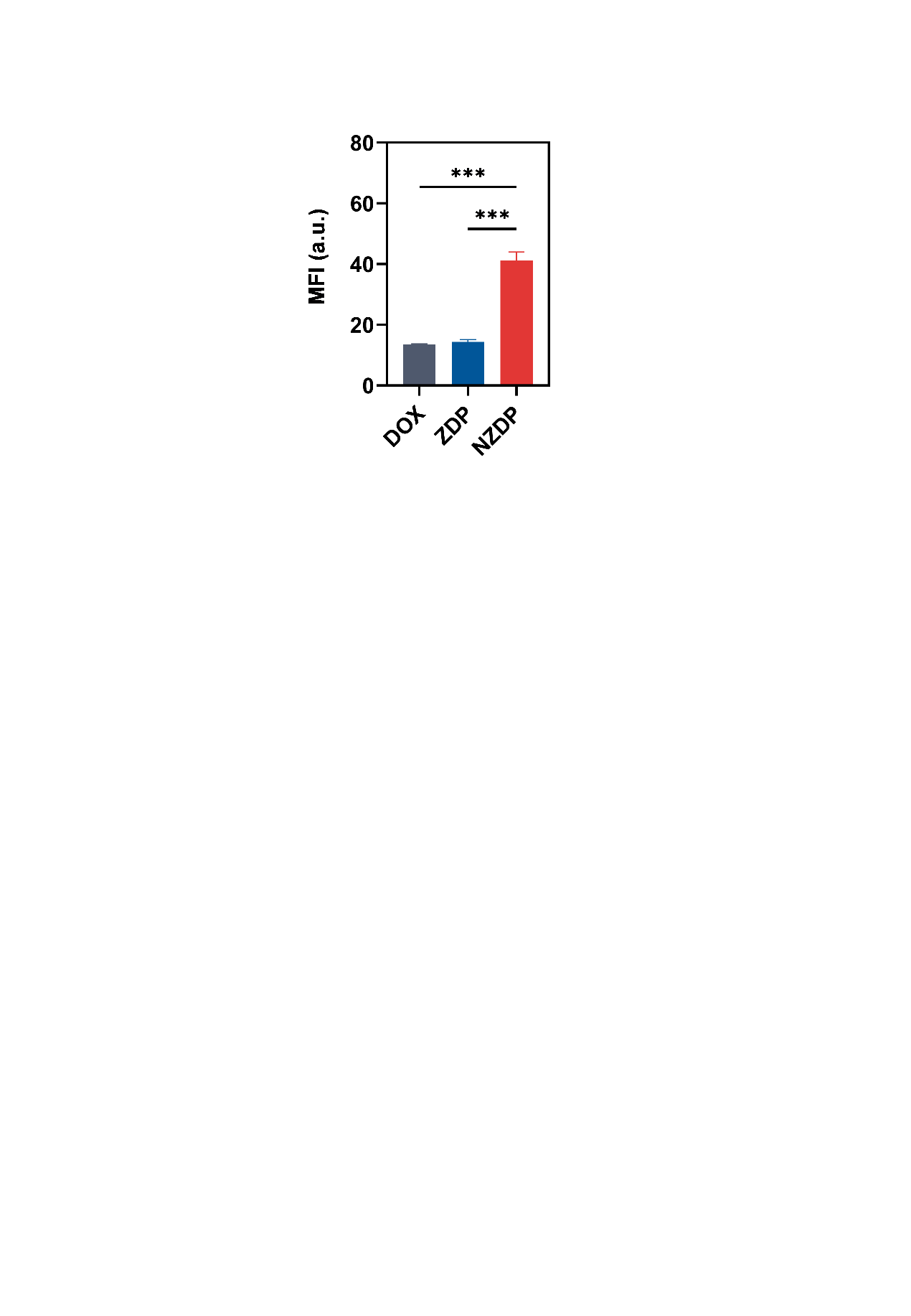


**Figure S7**. Fluorescence semi-quantitative analysis of free DOX, ZDP, and NZDP. n = 3; ****P*＜0.001.


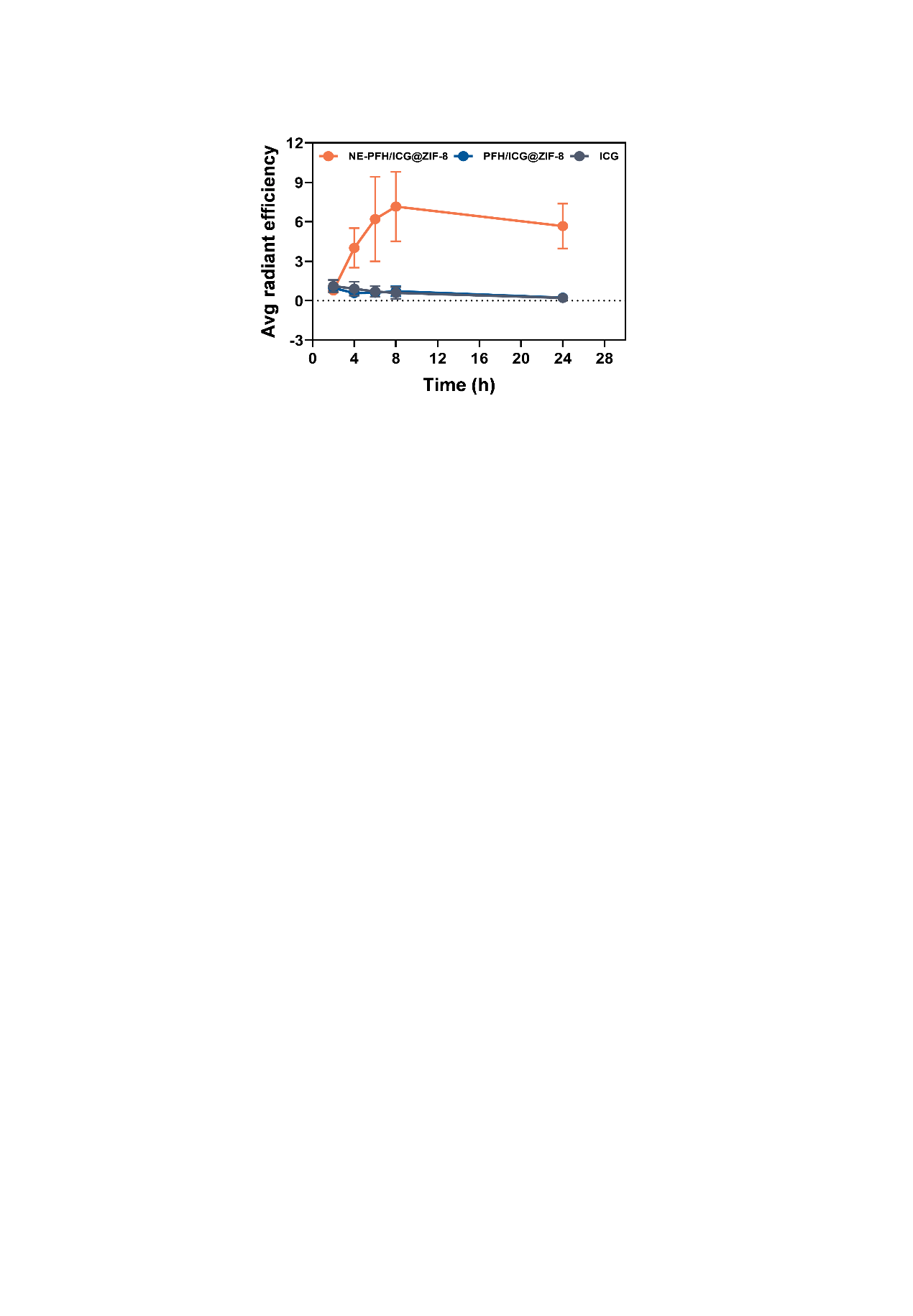


**Figure S8**. Quantitative analysis of the average fluorescence signal in the tumor area at different times. n = 3.


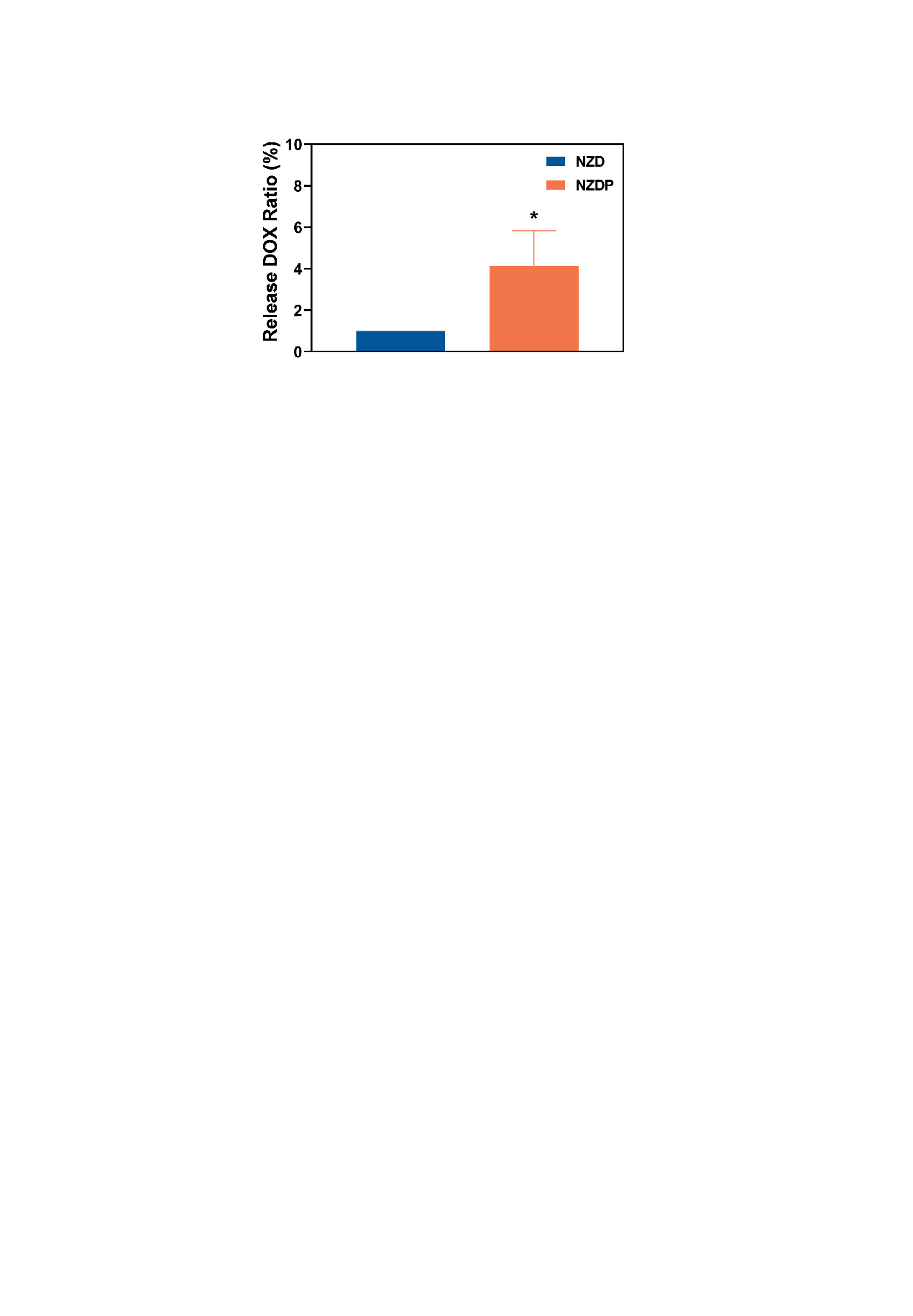


**Figure S9**. Effects of ultrasonic irradiation on drug release based on PFH. n = 3; **P*＜0.05.


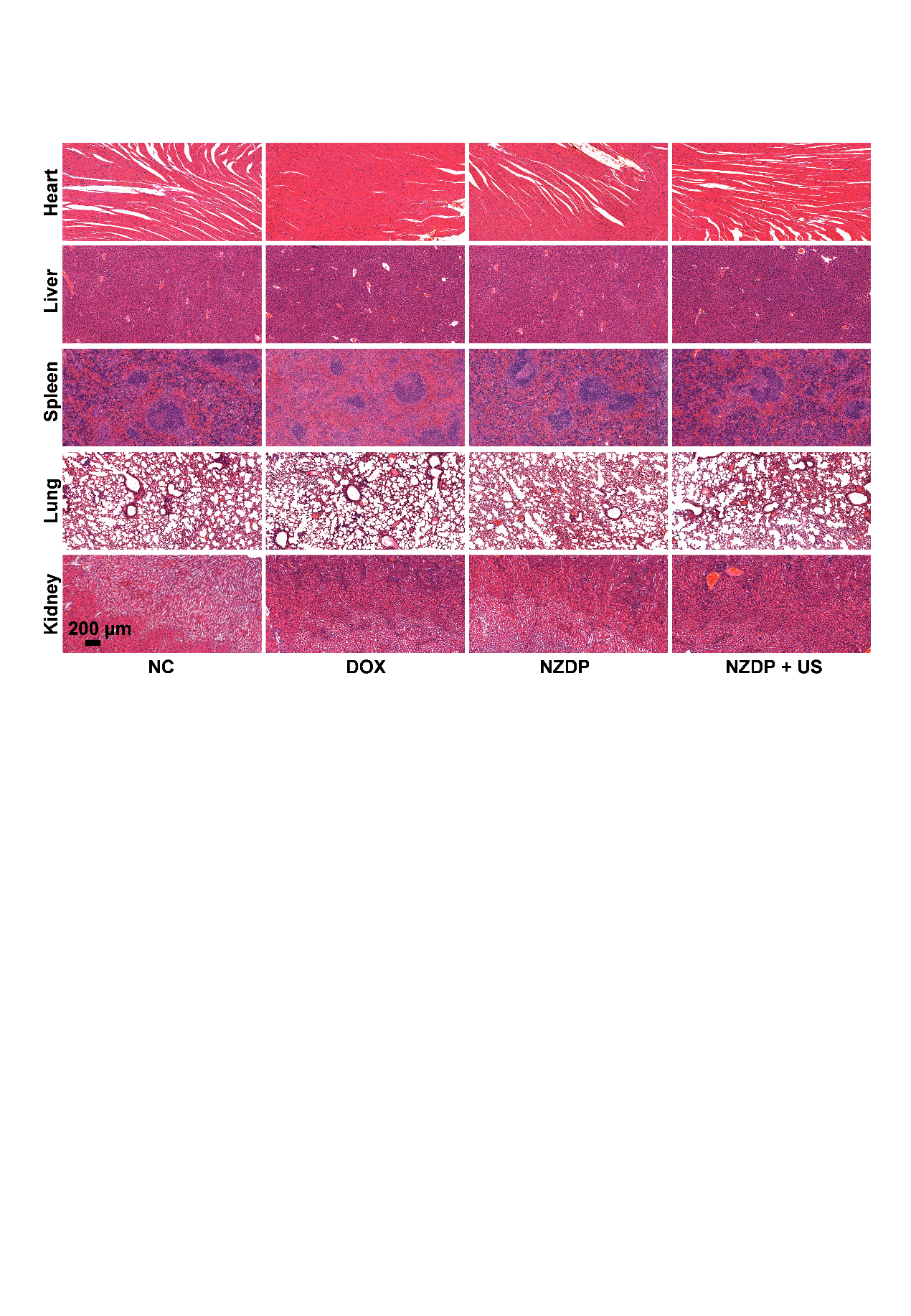


**Figure S10**. H&E staining of vital organs from mice after PBS, DOX, NZDP, and NZDP + US treatments. Scale bar: 200 μm.


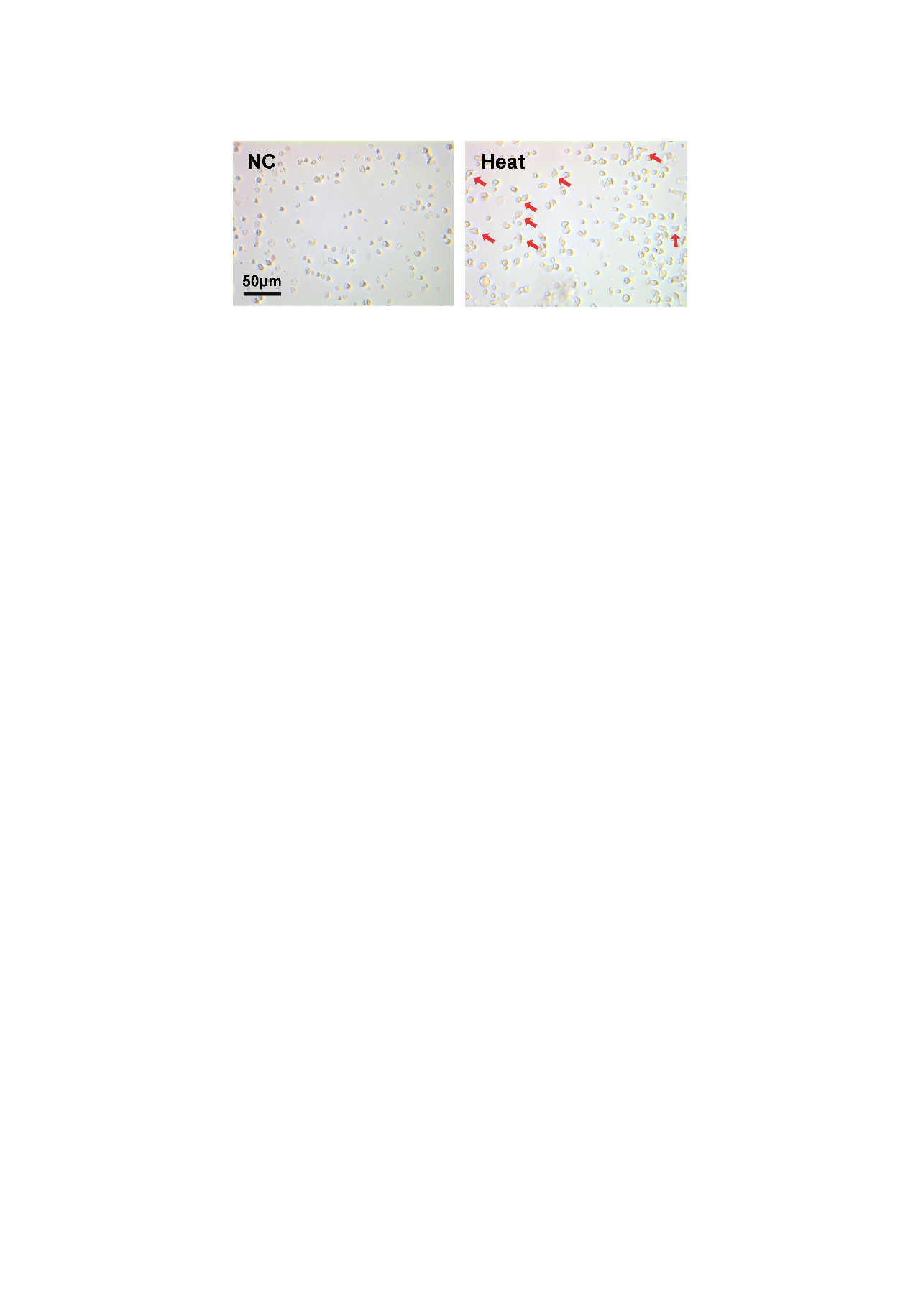


**Figure S11**. Morphological changes of NZDP after co-incubation with the supernatant of a sublethal heating model. Arrow: spindle-shaped NZDP, Scale bar: 50 μm.


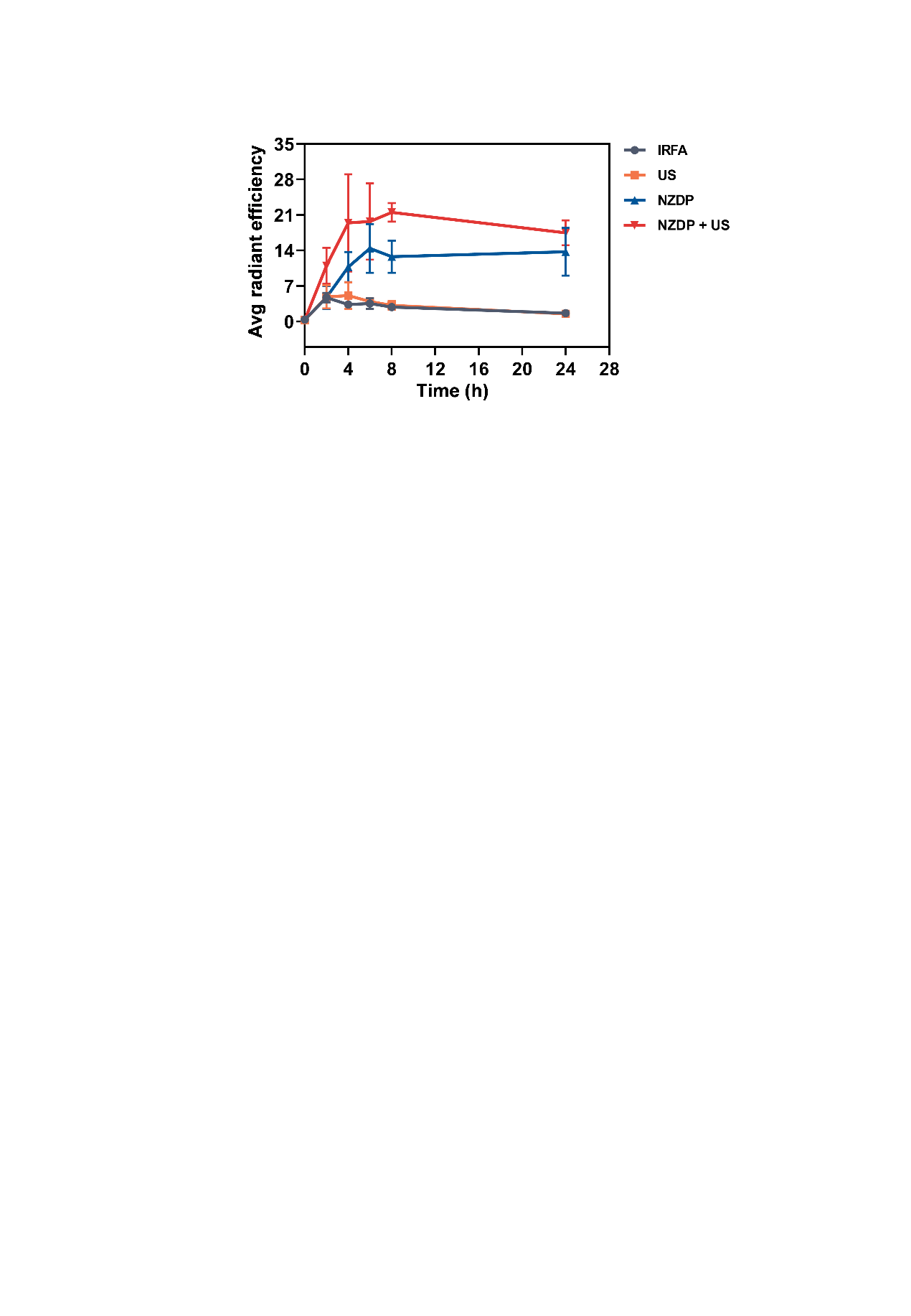


**Figure S12**. Quantitative analysis of the average fluorescence signal in the tumor area after IRFA, IRFA + US, IRFA + NZDP, and IRFA + NZDP + US at different times. n = 3.
